# Translational toxicology in setting occupational exposure limits for dusts and hazard classification – a critical evaluation of a recent approach to translate dust overload findings from rats to humans

**DOI:** 10.1186/s12989-015-0079-3

**Published:** 2015-04-23

**Authors:** Peter Morfeld, Joachim Bruch, Len Levy, Yufanyi Ngiewih, Ishrat Chaudhuri, Henry J Muranko, Ross Myerson, Robert J McCunney

**Affiliations:** Institute for Occupational Epidemiology and Risk Assessment of Evonik Industries, AG Rellinghauser Straße 1-11, Essen, 45128 Germany; Institute and Policlinic for Occupational Medicine, Environmental Medicine and Preventive Research, University of Cologne, Cologne, Germany; University Duisburg-Essen, Medical Faculty, Essen, Germany; IBE GmbH, Cologne, Germany; Cranfield University, ᅟ, Cranfield, UK; Orion Engineered Carbons GmbH, ᅟ, Cologne, Germany; Cabot Corporation, Billerica, MA USA; Muranko & Associates, Scottsdale, AZ USA; Department of Occupational Health, MedStar Washington Hospital Center, Washington, DC USA; The George Washington University School of Public Health, Washington, DC USA; Massachusetts Institute of Technology, Cambridge, MA USA; Brigham and Women’s Hospital, Boston, MA USA

**Keywords:** MAK, GBS, Granular biopersistent dusts, Poorly soluble dusts, OEL, Translational toxicology, Rat overload, Inflammation, Lung cancer

## Abstract

**Background:**

We analyze the scientific basis and methodology used by the German MAK Commission in their recommendations for exposure limits and carcinogen classification of “granular biopersistent particles without known specific toxicity” (GBS). These recommendations are under review at the European Union level. We examine the scientific assumptions in an attempt to reproduce the results. MAK’s human equivalent concentrations (HECs) are based on a particle mass and on a volumetric model in which results from rat inhalation studies are translated to derive occupational exposure limits (OELs) and a carcinogen classification.

**Methods:**

We followed the methods as proposed by the MAK Commission and Pauluhn 2011. We also examined key assumptions in the metrics, such as surface area of the human lung, deposition fractions of inhaled dusts, human clearance rates; and risk of lung cancer among workers, presumed to have some potential for lung overload, the physiological condition in rats associated with an increase in lung cancer risk.

**Results:**

The MAK recommendations on exposure limits for GBS have numerous incorrect assumptions that adversely affect the final results. The procedures to derive the respirable occupational exposure limit (OEL) could not be reproduced, a finding raising considerable scientific uncertainty about the reliability of the recommendations. Moreover, the scientific basis of using the rat model is confounded by the fact that rats and humans show different cellular responses to inhaled particles as demonstrated by bronchoalveolar lavage (BAL) studies in both species.

**Conclusion:**

Classifying all GBS as carcinogenic to humans based on rat inhalation studies in which lung overload leads to chronic inflammation and cancer is inappropriate. Studies of workers, who have been exposed to relevant levels of dust, have not indicated an increase in lung cancer risk. Using the methods proposed by the MAK, we were unable to reproduce the OEL for GBS recommended by the Commission, but identified substantial errors in the models. Considerable shortcomings in the use of lung surface area, clearance rates, deposition fractions; as well as using the mass and volumetric metrics as opposed to the particle surface area metric limit the scientific reliability of the proposed GBS OEL and carcinogen classification.

## Introduction

The term “translational toxicology” refers to the general approach of applying toxicological findings to human settings [1,2]. Here we use the term to describe the approach of using animal toxicology studies to conduct risk assessment and hazard classifications and to derive Occupational Exposure Limits (OELs). The latter is a quantitative application of animal data which goes beyond a qualitative translation in hazard assessment. Typically, No Observed Adverse Effect Concentrations (NOAECs) are determined in animal studies, and then adjusted by appropriate dosimetric and/or allometric modeling to perform a quantitative translation into Human Equivalent Concentrations (HECs). These exercises sometimes apply various conservative assumptions that in turn may result in very low “HECs” which are “deliberately” biased downward and are no longer equivalent e.g., [3]. While translational toxicology approaches have been around for many years [4-6], it is common to consider all available data including human epidemiology data when determining hazard classifications and OELs for potentially hazardous materials. In fact, if a robust epidemiological data set is available, then these results are typically given more weight in hazard classification and OEL development than animal toxicology studies [7].

In this paper, we analyze the scientific basis of a translational toxicology approach used by the German MAK Commission in their recommendations for exposure limits and carcinogen classification of “granular biopersistent particles without known specific toxicity” (GBS). Occupational exposure to inorganic dusts at concentrations less than current occupational exposure limits (OELs) can increase the risk of pulmonary disorders [8]. Particles once considered nuisance dusts and later “particles not otherwise classified” (PNOC) can cause and aggravate a number of pulmonary disorders, including chronic obstructive pulmonary disease (COPD) and asthma. A recent report noted that current OELs for these types of dusts, long considered inert, have been in place for over 30 years and are not fully protective against potential pulmonary damage [9]. The authors concluded that current exposure limits for these types of dusts need to be lowered. They recommended that on an interim basis *“safety and health professionals should consider 1 mg/m*^*3*^*of respirable dust as a more appropriate guideline than the value of 4 mg/m*^*3*^*currently used in Britain”* [9]. We note that this publication has been addressed by a Letter to the Editor [10].

Subsequently, the German MAK Commission issued an OEL recommendation for respirable GBS of 0.3 mg/m^3^ given a substance density of 1 g/cm^3^ [11]^a^. *“The threshold value does not apply for soluble particles, especially not for salts from rock salt and potash deposits, or for ultrafine (see Section Vh) or dispersed coarse particle fractions”* ([12], p. 197). To derive this proposed OEL, translational toxicology models, based on rodent data were applied and a number of conclusions were drawn related to the establishment of OELs and cancer classification for GBS including those currently regulated and those unregulated:

1. GBS cause lung cancer in rats due to chronic inflammation as a result of dust overload in the alveolar region of the lung.
2. If clearance mechanisms are not overwhelmed and, thus, inflammation is prevented, lung cancer risk will not be increased. Since excess lung cancers in the rat are only observed in conditions of lung overload, a threshold exists for adverse effects from exposure to these types of dusts. Thus, a NOAEC (no-observed adverse-effect concentration) exists; that is, a maximum concentration greater than 0, below which no adverse effects of GBS can be expected, including cancer.
3. The lung overload effect observed in rat inhalation studies is relevant for human risk assessment. Thus, a HEC (human equivalent concentration) exists that relates to the NOAEC, the maximum concentration that avoids lung overload in rats.
4. All GBS are carcinogenic to humans with a threshold effect (Carcinogen Category 4).

We emphasize that this cancer classification depends on the reliability of the translational toxicology models applied by the MAK Commission and discussed below in detail. The MAK commission stated: *“… the data obtained in test animals on the potential carcinogenicity of particles can be applied to humans if species-specific conditions (anatomy and histology of the respiratory tract) are taken into account”* ([11], p. 19). (see the MAK Committee’s manifesto on the carcinogenicity classification ([11], p. 63.)

The MAK recommendations are being considered by the Scientific Committee on Exposure Limits (SCOEL), an advisory group to the European Commission, which is now evaluating the potential use of these recommendations for European Member States [13]. In the light of the potential regulatory and policy implications of the MAK guidelines, we examined the scientific studies and assumptions that were used as the basis of the proposed exposure limit. Two fundamental approaches were chosen [11]: one based on retained particle mass per alveolar surface area (Model A) and another based on retained particle volume per macrophage pool volume (Model B, [14]) in rat inhalation studies, investigating the effect of dust exposure on inflammatory markers. These approaches were used to estimate the HEC. As a compromise between 0.15 mg/m^3^ and 0.25 mg/m^3^ (HEC according to Model A) and 0.5 mg/m^3^ (HEC according to Model B), an OEL was recommended for the respirable fraction of 0.3 mg/m^3^ for GBS with a particle density of 1 g/cm^3^. This value replaced the previous general dust limit for the respirable fraction of 1.5 mg/m^3^ [15]. Because the above considerations only apply to the respirable fraction, the former general dust limit for the inhalable fraction of 4 mg/m^3^ [15] remains valid.

The derived MAK OEL for respirable GBS is solely based on the quantitative translation of rat overload experiments into HECs without any consideration given to human epidemiological studies. This new MAK approach is a substantial departure from principles that have been used for many years in including results of human studies, most notably epidemiological investigations. To rely so heavily on translational toxicology models only, the new approach must be transparent, consistent, and evidence-based.

The purpose of our analyses is to review the recommendations for GBS by examining the scientific assumptions used by the MAK Commission. We have attempted to reproduce the derivations and recalculate the results by employing the translational toxicology methods used for Model A and Model B [14].

## Use of models as suggested by the MAK commission

The German MAK Commission [11] proposed two procedures (Model A and Model B) to estimate a no-observed adverse-effect concentration (NOAEC) for the respirable dust fraction of GBS in rats and to translate this value into a HEC. When we tried to apply the Models A and B we discovered a number of numerical problems. In the following Section we present, analyze and discuss Model A ([11], p. 54–58).

### Model A: The retained particle mass per alveolar surface area model

Model A assumes one lung compartment (alveolar) and a constant (species-specific) alveolar clearance rate below the overload effect. The input into the alveolar compartment is determined by the particle deposition rate in the alveolar region [mg/day]. The output from the alveolar compartment can be expressed as the particle burden in the alveolar region [mg] x alveolar clearance rate [1/day]. Given steady state we have input = output, i.e., deposition = steady-state burden x clearance rate. It follows that:

Steady state particle mass burden in the alveolar region [mg] = particle mass deposition rate in the alveolar region [mg/day]/alveolar clearance rate [1/day].

Particle mass deposition rate in the alveolar region [mg/day] was defined as dust concentration in inhaled air [mg/m^3^] x alveolar deposition fraction [%] x tidal volume [m^3^ per breath] x respiratory rate [breaths per day].

The alveolar deposition fraction was calculated for rats and humans by applying the MPPD (multiple-path particle dosimetry) Version 2.0 program (see for information on the MPPD: de Winter-Sorkina and Cassee [16] and http://www.ara.com/products/mppd.htm). The constant alveolar clearance half times are assumed to be 60 days for rats and 400 days for humans. Based on these assumptions and other input data, the steady state particle mass burden in the alveolar region is calculated for rats at the maximum exposure concentration when they showed no inflammation/overload according to Muhle et al. [17] who studied the effect of toner particles and pigmentary titanium dioxide (TiO_2_) particles: NOAEC_toner_: 1 mg/m^3^ respirable and NOAEC_TiO2_: 5 mg/m^3^ respirable.

These amounts of retained dust masses are the “numerator” of the metric in the particle-mass lung-surface model. When converting from rats to humans, the ratio of the “numerators” and the ratio of the alveolar lung surfaces are taken into account (ratio of “denominators”).

Model A is based on the metric “retained particle mass per lung surface area”. The working assumption in Model A is that the effect of the dust is species-independent, when described on the scale of the “retained particle mass per lung surface area” metric. The MAK Commission [11] states: *“that the sensitivity of the two species, rats and humans does not differ at the same dose/m*^*2*^*lung surface area”* ([11], p. 54). The footnote on page 56 of [11] clarifies that the numerator (i.e., the dose) is the retained particle mass and not particle volume or particle surface area: *“The inhaled particle concentration and the deposited/retained particle dose were determined as particle mass in the studies and were also included in the calculation as particle mass”*. This concept that a given mass of a substance acting on a unit area of lung tissue generates the same qualitative and quantitative effect in the lung across species is described as one possible application in Oller and Oberdörster [18], *Figure One: retained accumulated doses in μg/cm*^*2*^*yield similar health effects in rats and humans* (although not applied in this example to GBS but to soluble materials). The metric of Model A is mentioned additionally by a working group of the U.S. EPA: *“alveolar mass per alveolar surface area”* [19]. Based on this metric, the MAK Commission [11] used the rat experiment data of Muhle et al. [17] to derive HECs for toner and TiO_2_ by translational toxicology.

Model A leads to HECs of 0.133 mg/m^3^ (toner) and 1.02 mg/m^3^ (TiO_2_). Next, the MAK Commission applied a density division and derived a generic HEC (respirable) of about 0.15 mg/m^3^ to 0.25 mg/m^3^ for a substance density of 1 g/cm^3^ (0.133/1.2 = 0.11; 1.02/4.3 = 0.24).

We note that the rat NOAECs were 1 mg/m^3^ (toner) and 5 mg/m^3^ (TiO_2_). In light of the attained HEC values, this means that humans are more sensitive to inflammation/overload than rats on the mg/m^3^- exposure scale according to this calculation.

In the following, we present arguments and discuss the shortcomings of the Model A. In particular, we highlight the following^b^:

- The post-hoc density adjustment of the derived HEC values is inconsistent and not justified,
- The lung surface area values used in the calculations are not evidence based,
- The alveolar clearance rate chosen for humans can be disputed according to current research.

#### Model A: Inconsistent post-hoc density adjustment

Applying the “retained particle mass per lung surface area” metric, the MAK Commission [11] derived HEC values for toner and TiO_2_. After arriving at these values, a post-hoc correction of the derived HECs was performed by dividing the values with the respective densities of toner and TiO_2._ The Commission [11] states: *“The first derivation yields for toner a limit value of 0.133 mg/m*^*3*^*(density 1.2) and for titanium dioxide a value of 1.06 mg/m*^*3*^*(density 4.3) or for a density of 1, a value of 0.11 mg/m*^*3*^*for toner and 0.25 mg/m*^*3*^*for titanium dioxide”* (p. 63). We note that the units for the densities are omitted (e.g., “density 4.3” should read “density 4.3 g/cm^3^”). Thus, if the division is performed with units, one gets: 1.06 mg/m^3^/4.3 g/cm^3^ = 0.00000000025 (=2.5 × 10^−10^). Note that the units cancel out and the result has no units. Given that the final result of the derivation process should represent a concentration (HEC), it must be expressed with units, namely “mg/m^3^”. This is not the case with Model A, if values are computed correctly. In addition, we note that the correct value is 9 orders of magnitude lower than the MAK-derived value of 0.25 mg/m^3^ as given in [11].

In the following, we will show that the assumptions and equations of Model A imply that a density dependency of the NOAEC is logically ruled out. Thus, the post-hoc performed division of the HEC by the substance density contradicts the core principles of Model A. We explain this substantial problem in detail below.

The MAK Commission [11] calculated the alveolar deposition fraction of particles with the multiple-path particle dosimetry (MPPD) program, Version 2.0 (see for information on the MPPD: de Winter-Sorkina and Cassee [16] and http://www.ara.com/products/mppd.htm). Using the same version of the MPPD program as used in [11], we simulated a run where particle distribution and concentration are kept constant while substance densities are varied. The results shown in Table 1 demonstrate that the alveolar deposition fraction does not largely depend on the substance density if the same particle distribution and the same concentration are applied in two experiments with substances of different densities. We note that there is some marginal dependence, but this is in the opposite direction: substances with higher density have a somewhat larger deposition fraction, and not a smaller deposition fraction as would be needed to justify the density division performed in [11]. Conservatively, we assume in the following that the alveolar deposition fraction is independent of the substance density.

**Table 1:** **Alveolar deposition fractions calculated with MPPD, Version 2.0**

**Substance density/g/cm** ^**3**^	**Alveolar deposition fraction / %**
1	4.0
1.2	4.0
2	4.1
3	4.2
4	4.2
4.3	4.2
5	4.2

Results are shown for toner [17] with a true density of 1.2 g/cm^3^ and varied densities from 1 g/cm^3^ up to 5 g/cm^3^ while keeping all other input data constant. The range includes the density of TiO_2_, 4.3 g/cm^3^.

Next, we analyze the MAK Commission’s assumption on elimination (alveolar clearance). The basic equation (1) on page 54 in [11]

1 $$ \mathrm{steady}\ \mathrm{state}\ \mathrm{lung}\ \mathrm{load} = \mathrm{mean}\ \mathrm{deposition}\ \mathrm{rate}/\mathrm{clearance} $$

describes a one-compartment model with a constant clearance rate. A constant alveolar clearance rate means that per time unit, identical percentages of the deposited particles (number, mass, volume) will be eliminated from the alveolar region (we note that this means that equation (1) rules out an elimination of identical masses per time unit). This interpretation is in agreement with the term “elimination half time” used in [11]: see equation (4) on page 55. According to the findings described in Bellmann et al. [20], Muhle et al. [17] and Pauluhn [14], MAK applied identical elimination half times in rats of 60 days for toner and TiO_2_ despite the different densities of both substances (see for toner equation (5) on page 55 and the calculation for TiO_2_ on page 57 in [11]). The implication is that, besides the particle deposition fraction, particle clearance is also independent of “density”.

Given that identical masses are deposited (as we have shown in Table 1) and that the elimination half time of alveolar clearance does not vary with substance density as inferred in the use of an identical elimination half time in rats of 60 days for toner and TiO_2_ by the MAK Commission, equation (1) implies that identical masses will be retained for toner and TiO_2_. The effect metric of Model A is “retained particle mass per alveolar surface area“, meaning that equal masses of two different substances independent of their densities, acting per unit area of lung tissue should trigger the same effect. It thus follows that the HEC must be density independent. In conclusion, the post-hoc density correction of the HEC contradicts the basic assumptions of Model A.

The MAK Commission justifies the density correction in [11] as follows *“even though the dose deposited per m*^*2*^*lung surface area is calculated in procedure A, macrophage-elicited alveolar particle clearance has to be considered in the chronically retained particle dose; the particle density/particle volume is also relevant for this particle clearance in procedure A. Therefore, the particle density has to be taken into account in procedure A”* ([11], p.53). This rationale, however, implies that the effect metric has been changed and that the retained mass per surface area is no longer the only and most critical measure. Again, this conflicts with basic assumptions of Model A to translate the rat study findings to humans by a comparison of lung surface areas. Moreover, following the logic for density correction provided above, one should expect varying elimination half times for TiO_2_ and toner assuming a difference in the density dependent alveolar clearance rates between TiO_2_ and toner by a factor of 3.6 (because the ratio of the densities is 4.3/1.2 = 3.6). Experimental findings however report to the contrary [14,17,20].

Based on the arguments provided above, we conclude that the performed post-hoc density correction is unjustified and should be eliminated to achieve a consistent Model A.

#### Model A: Lung surface area values used by MAK are not derived using contemporary recognised standard procedures endorsed by the American Thoracic Society and the European Respiratory Society

Model A assumes that *“the sensitivity of the two species rats and humans does not differ at the same dose/m*^*2*^*lung surface area”* ([11], p. 54]). Thus, to translate findings from rat studies into HECs, the lung surface areas of both species have to be taken into account. MAK used *“an alveolar surface area of 57.22 m*^*2*^*for humans and 0.297 m*^*2*^*for rats”* ([11], p. 54). This leads to a surface area ratio (i.e. translation factor) of 57.22/0.297 = 193. These alveolar surface area values are taken from Table Five in Brown et al. [5]. The numbers were also reported in US EPA [21] but no longer in the updated document [22]. Unfortunately, the values presented in Brown et al. [5] cannot be reproduced because the basic data and the calculations are not given. Brown et al. [5] referenced Yeh et al. [23] who reported on *one* Long-Evans rat (not Fischer rats) and the authors referenced Yeh and Schum [24] who studied *one* man. First, we note that when studying the effects of toner and TiO_2,_ Muhle et al. [17] used Fischer rats (F344) in their research and this paper formed the basis of the MAK Commission’s HEC derivation in Model A. However, *“for interspecies dosimetric adjustments when translating animal toxicological study results obtained in rats to judge the potential for effects to be seen in humans, dosimetry calculations should be done using strain specific lung geometries”* [25]. Second, the method applied by Yeh et al. [23] and Yeh and Schum [24] (silicone rubber cast with added mathematical extrapolations) does not fulfil the conditions of the reference procedure defined jointly by the American Thoracic Society and the European Respiratory Society of how to measure lung surface areas [26]. The standard method, however, was applied in Gehr et al. [27] (on 8 humans) and in Stone et al. [28] (on 4 Fischer rats, i.e., the rat strain of interest). According to these standard procedure measurements, the best alveolar surface area estimates are 143 m^2^ (human) and 0.41 m^2^ (F344 rat), leading to a ratio of 349 = 143/0.41. We note that Stone et al. [28] reported on human surface areas also, but the measurements were based on surgically resected lung lobes (not an *in situ* instillation). Thus, an underestimation of the true surface areas is probable because of a post-mortem atelectasis and the best human data available are published in Gehr et al. [27]. An overview is given in Table 2.

**Table 2:** **Alveolar surface areas in rats and humans (the toxicological study** [17] **applied in model A used Fischer rats F344)**

	**Species/strain**	**N**	**Method and comments**	Lung function status*	**Alveolar surface area: absolute values**	**Alveolar surface area: ratio human/rat**	
Yeh [23]	Rat (Long Evans, 330 g)	1	silicone rubber cast (does not entail the alveolar region)	TLC	0.5725 m^2^ (mentioned as calculated value on p. 487, no value given in Table Two)	-	-
Yeh & Schum [24]	Human (60 years, ♂)	1	silicone rubber cast (does not entail the alveolar region)	TLC	No value given (neither in the text nor in Table Two)	-	-
US EPA [21]Brown et al. [5]	Rat (authors reference Yeh et al. [23], scaled to FRC) human (authors reference Yeh and Schum [24], scaled to FRC)	1	silicone rubber cast (does not entail the alveolar region) derivation of values in Brown et al. [5] cannot be reproduced from Yeh et al. [23] and Yeh and Schum [24] because of missing data and an unknown algorithm	FRC	0.2972 m^2^	193 (57.22/ 0.2972)	
		1		FRC	57.22 m^2^		
Gehr et al. [27]	Human (19 – 40 J)	8	in situ instillation with glutaraldehyde solution, evaluation by electron microscopy (evaluation according to the reference method, Hsia et al. [26])	TLC	143 m^2^ ± 12		349 (143 acc. to Gehr/0.41 acc. to Stone) (identical methods and F344 rats)
Stone et al. [28]	Rat (F344, 290 g)	4	in situ instillation with glutaraldehyde solution, evaluation by electron microscopy (evaluation according to the reference method, Hsia et al. [26])	TLC	0,41 m^2^ ± 0,04	249 (102,2/ 0,4)	
					0.4 m^2^ ± 0,03		
	Rat (SD, 363 g)	8					
	Human (nonsmoker)	4 (3♀, 1♂)	surgically resected lung lobes (no in situ instillation, underestimation probable because of post-mortal atalectasis)		102.2 m^2^ ± 20,5		

*TLC: total lung capacity, FRC: functional residual capacity.

The lung surface area data of Gehr et al. [27] are published in an often cited text book on comparative biology [29]. EU institutions recognise and refer to these values in their documents: *“over the huge alveolar surface area of 150 m*^*2*^*, the deposited particles are separated from the capillary blood by a tissue barrier”* ([30], p. 24). This value is also recognised by the International Commission on Radiological Protection (Human Respiratory Tract Model, [31]). MAK based the Model A calculations on the MPPD 2.0 deposition model. It is important to note that even the authors of the MPPD program used the lung surface area data of Gehr et al. [27] in their applications [16]. Thus, the results of Gehr et al. [27], obtained with standard procedures [26], are widely recognised and used as the reference for the human lung surface area. The MAK Commission dismissed the value from Gehr et al. [27] arguing that: *“the author himself points out that the true values may range between 70 and 100 m*^*2*^*”* ([11], p. 55). Apparently, the MAK Commission appears to have misinterpreted the work of Gehr et al. [27], who stated on page 136: *“we have shown on rat lungs that the ‘true’ alveolar surface available for gas exchange must be 25-50% smaller than the epithelial surface, depending on the level of air space inflation.… If this is taken into consideration the ‘true’ alveolar surface of the human lungs included in this study is reduced to 70–100 m*^*2*^*”.*

It is clear that Gehr and colleagues [27] discussed the surface available for gas exchange and not the epithelial surface. We note that the latter is relevant as the denominator in Model A’s metric. Furthermore, Gehr et al. [27] discussed the variation of the alveolar surface area in dependence on the air space inflation and Gehr’s argument relies on the assumption mentioned in [27] that the ratio between human and rat lung surfaces do not vary with air space inflation. Thus, the derived ratio of 349 remains valid irrespective of what degree of air space inflation is assumed to define ‘true’ values.

In summary, by using rat and human lung surface area data determined by internationally recognised methods, we have derived a translation factor of 349 which is about 1.8 fold higher than the MAK derived value of 193 [27,28]. We thus conclude that the MAK Commission [11] did not use the data best available on alveolar surface areas to derive the translation factor from Fischer rats to humans.

#### Model A: The alveolar clearance rate chosen for humans can be disputed according to current research

The applied value for the human alveolar clearance half time of 400 days is too large in comparison to current estimates of 255 days [32]. Current estimates were based on a two-compartment model that distinguishes the bronchiolar and interstitial region as target compartments of the alveolar region. The model was originally developed by Kuempel et al. [33] to predict lung and lymph node particle retention in US coal miners. Gregoratto et al. [32] adapted this model to amend the Human Respiratory Tract Model of the International Commission on Radiation Protection (ICRP) [31] and the authors validated this model with new human data from three studies of people exposed to radioactive aerosols, e.g. teflon particle and cobalt exposure. The model structure was recently adopted by the ICRP to describe the long-term particle clearance and retention of particles in the alveolar-interstitial region of the human respiratory tract [34]. The applied value of 400 days ignores a clearing of the particles from the human alveolar region into the interstitium, a critique also made by ECETOC ([34], Section 2.3, p. 17). Importantly, the comparative anatomical/histological study of Nikula and coworkers [35] on lungs of rat and human loaded with particulate matter corroborates the fundamental differences of clearance dynamics between both species (see also the Section on species-specific responses below).

In addition, a value of about 250 days follows from the general allometric scaling procedure proposed by West et al. [36]. We applied this independent approach based on allometric scaling to estimate the ratio of rat and human alveolar clearance rates (and half times). For extrapolation of the rat alveolar clearance rate k_rat_ to a human alveolar clearance rate k_human_, an allometric scaling according to West et al. [36] should yield

$$ {\mathrm{k}}_{\mathrm{human}} = {\mathrm{k}}_{\mathrm{rat}} \times {\left(\mathrm{bodyweigh}{\mathrm{t}}_{\mathrm{rat}}/\mathrm{bodyweigh}{\mathrm{t}}_{\mathrm{human}}\right)}^{1/4}. $$

Given a clearance half time of 60 days and a bodyweight of 250 g for the rat we derive as an estimate for the human alveolar clearance half time, assuming a weight of 70 kg: 60 days/[(0.25 kg/70 kg)^(1/4)^] = 245 days (we note that we discuss half times which are indirectly proportional to rates: half time = ln(2) / rate). This calculation, according to West et al. [36], supports the value of 255 days proposed by Gregoratto and colleagues [32]. If a larger bodyweight of 330 g is chosen for the rat, the estimated human half time will be 230 days.

#### Model A: Outcome on the HEC estimates

Assumptions made in a derivation process clearly affect the resulting value of any derived OEL. In this subsection, we want to demonstrate the range of values that can occur and how different these values are from the MAK-derived values when internationally standardized data for pulmonary clearance and lung surface area are used.

As described in the Section on lung surface area values above the ratio of the alveolar surface areas according to Gehr et al. [27] and Stone et al. [28] is 349. The Commission [11] applied a ratio of 193, which leads to a correction factor of 1.81 = 349/193. The alveolar clearance half time according to Gregoratto et al. [32] is 255 days. MAK [11] applied 400 days. This leads to a second correction factor of 1.57 = 400/255.

Using the correction factors for clearance rate and lung surface area, the following estimates for HEC derived with Model A are obtained:

$$ \mathrm{HE}{\mathrm{C}}_{\mathrm{toner}}=0.134\times 1.81\times 1.57\ \mathrm{mg}/{\mathrm{m}}^3 = 0.38\ \mathrm{mg}/{\mathrm{m}}^3 $$

$$ \mathrm{HE}{\mathrm{C}}_{\mathrm{TiO}2}=1.07\times 1.81\times 1.57\mathrm{mg}/{\mathrm{m}}^3=3.04\ \mathrm{mg}/{\mathrm{m}}^3 $$

These estimates should also apply for a substance density of 1 g/cm^3^ because the HEC is independent of density according to the fundamental assumptions of Model A. The large range of the HEC estimates seen above shows that Model A is not appropriate for deriving a generic OEL for all GBS.

### Model B: The retained particle volume per macrophage pool volume model

MAK’s Model B is another approach proposed to estimate a no-observed adverse-effect concentration (NOAEC) for the respirable dust fraction of GBS in rats and to translate the estimated NOAEC into a HEC. Model B is based on the publication of Pauluhn [14]. The specific metric of Model B - different from Model A’s metric - is assumed to be species-independent: the retained particle volume per alveolar macrophage pool volume [14].

Like Model A the retained particle volume per alveolar macrophage pool volume [14] model assumes one lung compartment and a constant (species-specific) alveolar clearance rate below the overload threshold. Because Model B focuses on the volume of the particles the units change from mg to μl (compare the Section on Model A): steady state particle volume burden in the alveolar region [μl] = particle volume deposition rate in the alveolar region [μl/day]/alveolar clearance rate [1/day].

The particle volume deposition rate in the alveolar region [μl/day] is calculated as dust concentration in inhaled air [mg/m^3^]/density [g/cm^3^] x alveolar deposition fraction [%] x tidal volume [m^3^ per breath] x respiratory rate [breaths per day]. We note that in contrast to Model A, the particle density is a necessary term in this equation, and HECs derived by Model B will be density dependent. We further note that the units are consistent: μl x g/cm^3^ = 10^−6^ × 10^3^ cm^3^ × g/cm^3^ = 10^−3^ g = mg.

It follows that a rat NOAEC in mg/m^3^ can be estimated as:

{steady state particle volume burden in the alveolar region [μl] x density [g/cm^3^] x alveolar clearance rate [1/day]}/{tidal volume [m^3^ per breath] x respiratory rate [breaths per day] x alveolar deposition fraction [%]}.

This justifies the structure of the important Equation (7) in [14], reproduced in [11] on page 59. Pauluhn [14] derived a factor of 1 to translate the rat overload NOAEC into a HEC (i.e., according to Model B rats and humans are of the same sensitivity on the mg/m^3^-exposure scale). Finally, Model B leads to

$$ \mathrm{H}\mathrm{E}\mathrm{C}\ \left[\mathrm{mg}/{\mathrm{m}}^3\right] = 0.5 \times \mathrm{particle}\ \mathrm{density}\ \left[\mathrm{g}/\mathrm{c}{\mathrm{m}}^3\right] $$

Model B is used in two derivations: the first relies on a Fortran program written by Prof. Dr. Jürgen Pauluhn, the second derivation can be performed without applying this program. The second derivation was used by the MAK Commission to derive an occupational limit value for all GBS ([11], p. 58–62). This volumetric approach has also been applied to biodegradable high molecular weight organic polymers [37].

We highlight the following^c^:

- The first derivation cannot be verified by an external reviewer because of unavailable information (i.e. the inaccessibility of the Fortran program),
- The standardization to rat lung mass or to rat body weight is varying and inconsistent,
- The deposition fractions applied cannot be reproduced with the cited MPPD program using the input parameters listed in [11],
- Assumptions used for the alveolar clearance rate for humans are incorrect and not based on the best available current research.

Below, we analyze the impact of these 4 points on the estimated HEC.

#### Model B: Non-replicable method applied for the calculation of the NOAEC in the first derivation

The first approach introduced in [14] describes the derivation of a volumetric NOAEC. This derivation relies on the Fortran program code with an unknown algorithm: *“A Fortran computer code was used for calculations”* ([14], Section 2.7, p. 182). The Fortran program was briefly described on page 142 in [38]. The code seems to estimate the daily increment of particle dose deposited in the alveoli using data from the multiple-path particle dosimetry model (MPPD, http://www.ara.com/products/mppd.htm) to calculate the fate of the deposited particles by applying elimination rate constants, and to superpose the resulting arrays to derive the retained particle burden. Because the code incorporates output data from the MPPD program, the derivation of the NOAEC based on the Fortran program likely suffers from the shortcomings detailed in Section on the deposition fractions below.

We reconstructed in Table 3 the chain of arguments as used in the first derivation and refer mainly to the 3rd paragraph of the Section on lung overload on page 181 in [14].

The respiratory volume of rats was calculated as 6 h × 60 min/h × 0.8 l/min/(day x kg-rat) = 288 l/(day × kg-rat) = 0.29 m^3^/(day × kg-rat). This number refers to a (theoretical) rat of 1 kg mass. Physiological dead space, assumed as 1/3 of the total inhaled volume, was used to estimate the alveolar ventilation volume of 0.19 m^3^/(day × kg-rat) on the basis of the respiratory volume: 2/3(0.29) m^3^/(day × kg-rat) = 0.19 m^3^/(day × kg-rat). Next, a critical particle volume in the rat’s alveolar space was calculated by the Fortran program code as 0.069 μl/day [see Figure Three in 14] (note that the caption to the figure confusingly uses different units: 0.069 μl/m^3^ - micro liters per cubic meter as opposed to micro liters per day**)**. The derived concentration in the rat’s alveolar volume of (0.069 μl/day)/(0.19 m^3^/day) = 0.36 μl/m^3^ was then used to calculate a corresponding respirable particle volume concentration in the inhaled air of 0.36 μl/m^3^ × 3/2 = 0.54 μl/m^3^. This figure represents the volumetric NOAEC, i.e., the maximum volume concentration that rats can inhale without becoming overloaded. *“The 2-year equivalent is 0.069 μl PM*_*resp*_*/0.19 m*^*3*^_*alv*_*or 0.36 μl PM*_*resp*_*/m*^*3*^_*alv*_*(see Figure Three). In terms of inhalation chamber concentrations and exposure durations (adjustment from alveolar ventilation to normal ventilation) this means that the above generic volumetric overload-threshold is attained when using daily exposure concentrations at …0.54 μl PM*_*resp*_*/m*^*3*^*for…chronic repeated inhalation exposures”* ([14], p. 181). Apparently, a factor of 3/2 was applied to convert PM_resp_/m^3^_alv_ to 0.54 μl PM_resp_/m^3^. This factor likely reflects a correction due to the deposition of the dust in the head and tracheo-bronchial region of the rat which was calculated using the MPPD Version 2.0. Applying the input parameters listed in the MAK document (including the “inhalabilty adjustment”, see ([11], p. 52) we calculated a deposition fraction in the head and tracheo-bronchial region of 25%. This would lead to a correction factor of 1/(1–0.25) = 4/3. In Pauluhn [14], a factor of 3/2. was used; MPPD 2.0 returned a deposition fraction of 1/3 (33%) if we choose the default option of the program and turned the “inhalability adjustment” to off. This would lead to an adaptation factor of 1/(1-1/3) = 3/2, which is identical to the value noted in Pauluhn [14].

We conclude that the calculations shown in Pauluhn [14] were performed with the default option of the program, without an application of an “inhalability adjustment”. Unfortunately, such important details are not documented in the publication and the author has not responded to our request for clarification (see Endnote 3). The “inhalability adjustment” is a recommendation made in the MAK document for calculating deposition fractions in rats with the MPPD program ([11] p. 58). Oller and Oberdörster [18] also made this recommendation.

We will analyze and discuss further problems encountered in replicating the deposition fractions applied in Model B calculations in more detail below (see the Section on the deposition fractions). We would like to emphasize, however, that the calculated value of 0.069 μl/day is a pivotal input to the first derivation of the NOAEC and relies solely on the unavailable Fortran code. Thus, it is unclear, if and how this analysis can be reproduced for verification.

#### Model B: The standardization by rat lung mass or rat body weight is varying and inconsistent

In Pauluhn [14] a second approach to estimate a NOAEC was suggested (see p. 181 and 182). Basic assumptions include:

- The number of alveolar macrophages was given as 6 × 10^7^/kg-rat and
- The volume of the alveolar macrophage of the rat as 1166 (μm)^3^ (see Table Two and the 3rd paragraph of the Section on lung overload on page 181 in [14]).

This leads to a volume of the alveolar macrophage pool of 6 × 10^7^ × 11.66 x10^−7^ μl/kg-rat = 70 μl/kg-rat [1 (μm)^3^ = 10^−18^ m^3^ = 10^−15^ l = 10^−9^ μl].

- Morrow’s original overload volume threshold [39] was set to 6% of the alveolar macrophage pool volume: 6% × 70 μl/kg-rat = 4.2 μl/kg-rat.
- The mass of the lung of a 330 g rat is given as 1.5 g in Table Five of Brown et al. [5], referring to Takezawa et al. [40]. Accordingly, Pauluhn [14] stated on p.181 *“4.5 g lung weight per kg-rat”*.

Thus, Morrow’s overload volume threshold can also be expressed as 4.2 μl/4.5 g-rat lung = 0.93 μl/g-rat lung, i.e., as a value of about 1 μl/g-rat lung.

To assess the corresponding overload concentration threshold in the chamber air (NOAEC), this second approach took into account the alveolar deposition fraction in rats calculated by MPPD and assuming an equilibrium (steady state) of deposition and clearance: see Equations (6) and (7) on pages 181 and 182, respectively in [14]. Equation (7) is of major importance because it yields the NOAEC, called NO(A)EL (predicted) in [14]. This equation is reproduced in [11] on page 59:

**Table 3:** **First volumetric approach to derive a NOAEC in rats (3rd paragraph of the section on lung overload on page 181 in Pauluhn** [14]**), all data are relative to a rat mass of 1 kg**

**Ventilation volume per day**	**Dead space fraction**	**Alveolar ventilation volume per day**	**Critical particle alveolar volume per day**	**Critical particle volume concentration in the alveolar space**	**Dust deposition fraction in the head and in the tracheo-bronchial region**	**Critical particle volume concentration in the inhaled air: NOAEC**
0.29 m^3^	1/3	0.19 m^3^	0.069 μl	0.36 μl/m^3^	1/3	0.54 μl/m^3^
		=		=		=
		2(0.29)/3 m^3^		0.069 μl/0.19 m^3^		3(0.36)/2 μl/m^3^
		Dead space correction	Output *Fortran* program		Setting the “inhalability adjustment” *off* in MPPD 2.0*	Correction for the dust deposition in head and tracheo-bronchial region

*Input data to MPPD V2.0: MMAD = 1.8 μm, GSD = 2, density = 1 g/cm^3^; particle characteristics according to Pauluhn [14], all other MPPD input parameters as listed in the MAK document ([11], p. 57,58 and Appendix).

$$ \mathrm{NO}\left(\mathrm{A}\right)\mathrm{EL}\left(\mathrm{predicted}\right)=\frac{1\ \mu l}{0.29\ {m}^3}\mathrm{x}\frac{\rho }{fvi}\mathrm{x}\frac{100}{PMresp}\left[\frac{\mathrm{mg}}{m^3}\right] $$

Equation (7) starts with the fraction 1 μl/0.29 m^3^. We note that 1 μl represents Morrow’s overload volume threshold expressed as 1 μl per g-lung whereas 0.29 m^3^ means the respiratory volume of 0.29 m^3^ per kg-rat. Obviously, units are confused and Morrow’s overload threshold should also refer to a 1 kg rat and, thus, should have been set to 4.2 μl per kg-rat in Equation (7). This correction increases the estimated NOAEC by a factor of 4.2.

#### Model B: Deposition fractions applied in Pauluhn [14] cannot be reproduced with the MPPD program given MAK’s input data [11]

Equation (7), p. 182 ends with the term 100/PM_resp_., which is the inverted alveolar deposition fraction because PM_resp_ denotes the deposition fraction in %.This notation is confusing: the author wrote in other places “F_a_ = fractional deposition of PM in the alveolar region, PM_resp_, as estimated by MPPD2 calculations” (p. 181/182 and 186) so that F_a_ is the deposition fraction and PM_resp_ means the “pulmonary deposited dose ‘PM_resp_’… estimated by MPPD2 calculation” (p.182). Nonetheless, the alveolar deposition fraction is of importance in the estimation of the NOAEC and this fraction was set to 7.5% ([14], p. 186). The MAK Commission [11] stated that Pauluhn [14] and the MAK Commission determined the alveolar deposition fractions with the help of MPPD Version 2.0. We applied this program version but calculated a fraction of 6.3%. According to the current MPPD Version 2.11 it is even lower, only 3.3%. To perform these calculations, we used input data taken from [14] (mass median aerodynamic diameter: MMAD = 1.8 μm, geometric standard deviation: GSD = 2, particle density = 1 g/cm^3^, p. 186) and the MPPD parameters as published in Table Four of Oller and Oberdörster [18] and, accordingly, in the MAK document [11]. Thus, it is unclear why a value of 7.5% was reported in [14] (Equation 8) and ([11], p. 61). Even larger problems arose when we tried to reproduce the alveolar deposition fraction in humans of 16.4% as applied in [14] (Equation 8) and by the MAK Commission ([11], p. 61): we arrived at a value of 8.4% with MPPD Version 2.0 and 8.8% with the current MPPD Version 2.11. Likewise, we could not replicate the substance specific deposition fractions in Table One of [14]. These problems also affect the adaptation factor AF_lung burden_ because it entails the ratio of the rat and human alveolar deposition fractions (see Equation (8) on p. 186 in [14]). For a more complete discussion and a potential explanation of the differences see the Section on our sensitivity analysis below.

We emphasize that a revision of the deposition calculations in [14] and [11] is needed because the deposition fractions were calculated with an MPPD version (i.e. MPPD Version 2.0) that is outdated. Hence the calculations of the MAK Commission are not based on a state of the art technique. We note that the outdated MPPD Version 2.0 is no longer publicly accessible to enable an independent reviewer to reproduce the results. Fortunately, one of the co-authors of this review has a copy of the outdated version which we used for our calculations.

#### Model B: The alveolar clearance rate chosen for humans is incorrect and needs revision based on recent research

The applied value for the human alveolar clearance half time of 400 days is too large in comparison to current estimates of 255 days [32]. The value of 400 days ignores a clearing of the particles from the human alveolar region into the interstitium, a critique also made by ECETOC ([34], Section 2.3, p. 17). In addition, a value of about 250 days follows from the general allometric scaling procedure proposed by West et al. [36], (see the Section on alveolar clearance rates in Model A above).

#### Model B: Sensitivity analysis: Deposition fractions and NOAECs

We evaluated the impact of some of the issues identified above and present the findings in Table 4. Only the second derivation, based on Equation (7), ([14], p. 182), can be analyzed because we could not obtain the Fortran program to do a sensitivity analysis of the first NOAEC derivation. We note that Equation (7) implicitly used an alveolar clearance rate in rats of 0.01/day that is not indicated. This is confusing because the equation should show all variables that have to be taken into account to evaluate Equation (7).

The first line of Table 4 repeats the calculations with input values as noted in [14]. The calculated NOAEC, the calculated adaptation factors and the overall finding of an estimated HEC = 0.53 mg/m^3^ agree with the results shown in [14]. One striking difference in all calculations, however, is the lower alveolar deposition fraction in humans: 16.4% in the first line but values between 8% and 9% in all other scenarios. The reason for this discrepancy is one different MPPD input parameter value: “Oronasal-Normal Augmenter” is chosen to characterize the breathing pattern in humans in all lines but the first where we used “Oronasal-Mouth Breather”. We emphasize that “Oronasal-Normal Augmenter” is a recommendation made in the MAK document ([11], p. 58 and Appendix). Indeed, for humans, the MAK Commission has published in other applications alveolar deposition fractions that are similar to our values (Table 4): 7.01% for toner dust and 8.72% for TiO_2_ dust using the substance data as given in Muhle et al. ([11], p. 56, 57, 17). Furthermore, the recommendation

**Table 4:** **Sensitivity of results in dependence on modified input data (MPPD input parameters, steady state particle volume, clearance half time in humans) and different MPPD program versions: Estimated NOAEC in rats, calculated adaptation factors AF**_lung burden_ **and AF**_clearance_ **, and the derived human equivalent concentration (HEC) according to the second Model B procedure described by Equation (7) on p.182 and Equations (8), (9), (10) on p. 186 in** [14]

**MPPD Version**	MPPD input parameters*	**Alveolar deposition fraction in rats /%**	**Alveolar deposition fraction in humans/%**	**Critical steady state particle volume burden/μL per kg-rat**	**Alveolar clearance half time in humans/days**	**NOAEC/mg/m** ^**3**^	**AF**_lung burden_ **/1**	**AF**_clear ance_ **/1**	**HEC/mg/m** ^**3**^
2.0	IA switched off OMB	7.5	16.4	1	400	0.53	0.93	0.93	0.53
2.0	IA ONA	6.3	8.4	1	400	0.63	1.52	0.93	1.03
2.11		3.3	8.8	1	400	1.21	0.76	0.93	0.98
2.0	IA ONA	6.3	8.4	4.2	400	2.66	1.52	0.93	4.33
2.11		3.3	8.8	4.2	400	5.07	0.76	0.93	4.14
2.0	IA ONA	6.3	8.4	1	250	0.63	1.52	0.58	1.65
2.11		3.3	8.8	1	250	1.21	0.76	0.58	1.58
2.0	IA ONA	6.3	8.4	4.2	250	2.66	1.52	0.58	6.93
2.11		3.3	8.8	4.2	250	5.07	0.76	0.58	6.62

*IA, Inhalability Adjustment; OMB, Oronasal-Mouth Breather; ONA, Oronasal-Normal Augmenter; Other input data used invariantly and in accordance with [14] and [11]: reference body weights = 1 kg-rat, 70 kg-human; ventilation rates = 0.29 m^3^/kg-rat and day, 10 m^3^/70 kg-human and day; macrophage pool volumes = 7x10^10^ μm^3^/kg-rat and 50x10^10^ μm^3^/kg-human; alveolar clearance half time in rats = 60 days; MMAD = 1.8 μm, GSD = 2, density = 1 g/cm^3^ (particle characteristics); other MPPD input parameters as listed in the MAK document ([11], p. 57, 58 and Appendix).

“Oronasal-Normal Augmenter” agrees with the tutorial text of MPPD 2.11. The tutorial states*: “Choose Oronasal-Normal Augmenter so as to perform the calculation for oronasal breathing for the normal case for which nasal breathing occurs under minute volumes of 35.3 L/min, but switches to combined nose and mouth breathing above this value”* (MPPD 2.11 Tutorial 2: Monodisperse for Human, http://www.ara.com/products/mppd.htm). Oller and Oberdörster [18] have also made this recommendation. In contrast, in Pauluhn [14] the breathing pattern chosen was “oronasally breathing humans” (p. 186). When we, however, interpreted this as the program option “Oronasal-Mouth Breather”, MPPD 2.0 returned a deposition fraction of 16.4% identical to that reported in [14]. We would like to note that MPPD 2.11 gave a similar value of 15.7% when choosing “Oronasal-Mouth Breather”, and that the human deposition fractions are comparable when calculated with MPPD Version 2.0 or 2.11. The MPPD Help function clarifies on Oronasal-Mouth Breathers: *“Habitual Mouth breathers are considered to breathe through the nose and mouth simultaneously, even at rest”.*

We conclude that Pauluhn [14] probably chose “Oronasal-Mouth Breather” to characterize the breathing pattern in humans, instead of “Oronasal-Normal Augmenter”, the recommendation of MAK and MPPD. Thus, the calculations in [14] seem to suffer from a divergent setting of MPPD input parameters on breathing patterns in humans. Furthermore, the MPPD 2.0 applications in Model B, as performed by the MAK Commission [11], are inconsistent and confusing.

Another noteworthy difference among the programs is the lower (~50%) alveolar deposition fraction in rats of 3.3% calculated by the current program (MPPD 2.11) in comparison to 6.3% (MPPD 2.0). This figure reflects true differences between program versions because the findings are based on identical input data. We can reproduce the value of 7.5% published in Pauluhn [14] if we switch off the “inhalability adjustment” in MPPD 2.0. The MPPD tutorial explains: *“Choose whether the program should adjust for inhalability of the aerosol using logistic functions suggested by Menache et al. [*41*] for small laboratory animals. For small particles, this inhalability is unity. By default, adjustment for inhalability is turned off.”* (MPPD 2.11 Tutorial 1: Monodisperse for Rat, http://www.ara.com/products/mppd.htm). We surmise that the default option of the program was used in Pauluhn [14] although use of the “inhalability correction” has been recommended by Oller and Oberdörster [18] and it is listed by the MAK Commission as the option to choose ([11], p. 58). Again, input data and results published by the MAK Commission in [11] are confusing and the program version applied is outdated. The entry “Check” for inhalability adjustment instead of “Yes” or “No” on page 78 in [11] adds to this confusion.

#### Model B: Sensitivity analysis: Outcome on the HEC estimate

Table 4 presents the following conclusions on derived human equivalent concentrations. A higher HEC of 1 mg/m^3^ is estimated if we apply the alveolar deposition fractions based on the MMPD input parameters as listed by the MAK Commission [11] and Oller and Oberdörster [18] (calculated with MPPD Version 2.0 or MPPD Version 2.11). We obtain a HEC estimate of about 1.5 mg/m^3^ when using the alveolar clearance half time in humans of 250 days as suggested by Gregoratto et al. [32]. If the overload threshold (critical steady state particle volume burden) is modified from 1 μl/kg-rat to 4.2 μl/kg-rat, all estimates are increased by an additional factor of 4.2. Thus, according to our calculations, the estimated HECs range from 1 mg/m^3^ to 7 mg/m^3^. Even if the alveolar clearance half time of 400 days is used, as proposed in Pauluhn [14] and by the MAK [11], the best HEC estimate is 4 mg/m^3^ for a substance with a density of 1 g/cm^3^, which is considerably higher than the 0.5 mg/m^3^ value derived in [14] and [11]. Because of these rather high HEC values and the large variation of almost an order of magnitude, the Model B approach appears to be of little value in general respirable dust OEL assessments.

## Commentary on the models

We discovered that the metrics of Models A and B are of dubious validity because of conflicting data. To provide another perspective on translating animal results to humans, we examine the plausibility of the metrics chosen, key epidemiological studies among worker cohorts exposed to GBS and human studies in which (bronchoalveolar lavage, BAL) results are available. We note that Model A’s focus on alveolar surface area can be criticized as it relies on a mode of action that is predominantly related to acute effects whereas, the main interest is in the chronic effects of GBS exposure mediated by macrophages [42].

### The particle mass and volume metrics in comparison to the particle surface area metric

The justification for the particle volume based approach used in MAK’s Model B given in Pauluhn [14] refers to Pauluhn [38] where is it is stated that *“the key metric of dose is particle mass and not particle surface area”* [38]. Pauluhn [38] studied ALOOH (aluminum oxyhydroxides = boehmite with primary particle diameters of 10 nm or 40 nm) and Fe_3_O_4_ (pigment-grade iron oxide = magnetite) in Wistar rats. We note that the retained particle mass is used as the numerator in the effect metric of MAK's Model A but not in Model B which is the model recommended in Pauluhn [14].

The author demonstrated (Figures Six, Seven and Eight in [38]) that markers for pulmonary effects in the lungs (e.g., polymorphonuclear leukocytes (PMN) cell counts) correlated well with retained particle burdens expressed as mass but poorly when the exposure was expressed in terms of particle surface area. Although evidence was provided for the particle mass metric, the author did not dismiss the importance of the particle volume based metric. *“However, due to the difficulty to reliably estimate PM volumes from aggregated PMs with different densities, polydisperse particle sizes, and void-spaces of packed particles within macrophages, volumetric estimates, although considered to be mechanistically important … were not considered in this analysis”* [38]. This issue is complicated further as the surface area using the BET (Brunauer, Emmett, and Teller) methodology *per se* [43] is not necessarily a unique characteristic of a particle. For instance, the specific surface area (N_2_ used as adsorbent) of AlOOH-40 nm, after drying and degassing (100°C at 0.1 mbar for 16 h) was 46.3 m^2^/g while under other conditions of measurement (550°C for 3 h) the BET was reported to be 105 m^2^/g. In Pauluhn [38] the larger value was applied for analyses. It would have been of interest to see how sensitive the reported findings are to the different BET measurement values. The validity of both approaches, Model A’s *particle mass metric* and Model B’s *particle volume metric*, appears in doubt based on a study of Tran et al. [44]. Male Wistar rats were exposed to aerosols of TiO_2_ and BaSO_4_ each at two separate concentrations: 25 and 50 mg/m^3^ for TiO_2_, and 37.5 and 75 mg/m^3^ for BaSO_4_. Duration of exposures were set to 209 days, 118 days, and 203 days and 119 days, respectively. The mass burdens of TiO_2_ and of BaSO_4_ were determined in lung and lymph-node tissue ([8], p. 1091–1093) and “*lung mass burdens were reexpressed in terms of total surface area of deposited particles. The specific surface areas of the two dusts were measured using Brunauer Emmett Teller (BET) gas adsorption at Morgan Materials Technology (Stourport-on-Severn, UK) from a sample of approximately 5 g of each dust*” ([43], p. 116). Tran et al. [44] reported that BaSO_4_ and TiO_2_ particles, of similar substance density (BaSO_4_: 4.5 g/cm^3^, TiO_2_: 4.25 g/cm^3^), showed different inflammatory responses across the retained particle *mass* and retained particle *volume* scale but had similar responses on the retained particle *surface area* scale. The authors concluded on p. 1117: *“The results presented here show that the total surface area of particles in the lung may be the dominant measure when quantifying the toxicity of poorly soluble “nuisance” or PNOC [particulates (insoluble) not otherwise classified] dusts. The strength of the evidence lies in the consistency of the relationships between both PMN and lymph-node burdens, measured on independent sets of rat lungs, and total surface-area burden.”* These relevant findings on the role of particle surface area were not discussed in [14,38]. We further note that Monteiller et al. [45] was cited in [38] as support of the particle volume based approach–but this interpretation diverges from the views of Monteiller et al. [45]. In their discussion of low-solubility low-toxicity particles (LSLTP) they wrote: *“These in vitro data support the contention that … surface area is the dose metric that relates best to the inflammatory response for a range of LSLTP. This finding accords with our previous in vivo studies, in which surface area of LSLTP was found to be the factor driving the inflammatory response in rats”* ([45], p. 614]). This UK working group came to the same conclusion in a review on appropriate dose metrics for poorly soluble dusts [46]. Other authors concur with this finding [47,48].

Oberdörster et al. [49] presented strong evidence in favor of the retained particle surface area concept and evidence against the retained particle mass and retained particle volume concepts by comparing nanostructured and pigmentary TiO_2_ in a 12-week inhalation exposure study in rats. The concentrations were 23.5 ± 2.9 mg/m^3^ for the nanostructured material and 22.3 ± 4.2 mg/m^3^ for the pigmentary TiO_2_. Upon aerosolization, both TiO_2_ particle types formed agglomerates with mass median aerodynamic diameters of 0.71 μm (nanostructured) and 0.78 μm (pigmentary) and with geometric standard deviations of 1.9 and 1.7, respectively. Since the aerodynamic diameters of the aerosols were essentially the same for the two particle types, the compartmental deposition in the respiratory tract of the animals was expected to be very similar. The authors concluded on p. 177: *“Neither average gravimetric nor average volumetric burdens of both particle types correlate well with the observed effect on AM clearance function. Expressing these doses as the retained particle surface area in the macrophages shows that the effects on AM-mediated clearance function of the two different particle types can be expressed by a common dose–response curve”*. Bermudez et al. [50,51] confirmed these findings. Lison et al. [52] concluded that, when conducting studies to elucidate the effect of particles on the lung, it is important for insoluble particles such as manganese dioxide to consider the administered dose in terms of surface area (e.g. m^2^/kg) rather than in gravimetric terms (e.g. mg/kg). ECETOC [34] summarized arguments in favour of particle surface area as the driving metric (Section 2.1.3, p. 10 and 11). Kuempel et al. [53] judged that the appropriate metric to evaluate effects of poorly soluble, low-toxicity particles is surface area dose of respirable particles. Cherrie et al. [9] suggested that the ideal OEL would be based on surface area of dust per unit volume of air inhaled “(e.g. cm^2^/m^3^)”. Saber et al. [54] described in their Figure Five the appropriate effect metric as a non-linear association between particle surface area and neutrophil influx (PMNs) showing a NOAEC. Johnston et al. [55] reviewed the particle attributes and biological mechanisms responsible for the observed toxicity *in vivo* and *in vitro* studies of silver and gold particulates. They concluded that differences in toxicology of smaller and larger particles are likely to be driven by differences in particle surface area, when administered at an equal-mass dose. Hext et al. [56] reviewed animal studies performed with TiO_2_ and concluded that the observed responses were consistent with the particle surface area of the lung burden. Braakhuis et al. [57] reviewed and analyzed published data on inhalation of nanoparticles to identify and evaluate physicochemical characteristics of nanoparticles that affect the development of pulmonary inflammation. Nanomaterials differ in their capacity to induce lung inflammation; no unifying dose metric could be identified to describe pulmonary inflammation for all nanomaterials. Surface reactivity appeared to have the best correlation with pulmonary inflammation. Simko et al. [58] proposed as dose metric “*the total deposited NP [nano particles] surface area (SA), which has been shown frequently to determine toxicological responses e.g. of lung tissue*”. Further applications and discussions arguing in favour of the retained particle surface area concept are given in Duffin et al. [59] and Donaldson et al. [60]. Maynard and Kuempel [61] performed an overview and concluded from their Figure Three that “*despite the varying particle compositions, sizes and morphologies, the aerosol surface area dose–response relationship appears to be remarkably similar for poorly soluble, low toxicity (PSLT) particles*”.

In contrast to these researchers, the particle volume approach is favoured in [42] as outlined in [14]. Although the work of Tran et al. [44] is cited in these publications, the importance of their findings are not discussed. The relevant study of Oberdörster et al. [49] was not described in Pauluhn [42], which is unfortunate because both studies [44,49] fulfill the conditions of informative experimental studies adopted by the author. We like to emphasize that Figure Eleven in [14] confirmed the problems Oberdörster et al. [49] had identified with the volume metric. All data points showing results for non-nanostructured materials with lower particle surface area are below the curve in Figure Eleven, and all data points for nanostructured materials with higher surface area are above the curve (only exception: Boehmite, ALOOH). Thus, there is no common response curve of the alveolar clearance rate of low and high surface area dusts across the volumetric dose as shown before by Oberdörster et al. [49]. A recent review [62] seemed to misinterpret the carbon black study by Elder et al. [63] as some evidence in favor of the volumetric approach. We note that such a view contrasts with the authors’ interpretation who stated in the abstract that “*the results from rats also show that particle surface area is an important determinant of target tissue dose and, therefore, effects*”. In this sub-chronic rat study with 13 weeks exposure time, the authors applied Printex 90, a high surface area carbon black, at 1 mg/m^3^, 7 mg/m^3^, and 50 mg/m^3^. In addition, they performed a 13 week rat experiment with Sterling V, a low surface area carbon black, at 50 mg/m^3^. First, according to the design of Elder et al. [63] a concentration of 7 mg/m^3^ Printex 90 should produce about the same surface area concentration as 50 mg/m^3^ of Sterling V. The authors found that Printex 90, 7 mg/m^3^ showed less relative and absolute PMN cell numbers than Sterling V. Second, if a particle mass based metric were correct we expect to see similar results when applying Printex 90 and Sterling V at the same concentration of 50 mg/m^3^. Given an identical density of Printex 90 and Sterling V we also expect similar results based on the particle volume model. We note that Printex 90 (50 mg/m^3^) showed more pronounced reactions, in particular when comparing absolute PMN cell numbers and when evaluating PMN findings after the longest post-exposure recovery time period of 11 months. Given these findings we agree with Elder et al. [63] who stated that the effect of Sterling V was often between the effects of Printex 90 (7 mg/m^3^) and of Printex 90 (50 mg/m^3^). The authors speculated that other surface properties of Sterling V may have amplified the effect of this low surface area carbon black, particularly they mentioned the higher PAH (polycyclic aromatic hydrocarbon) content of Sterling V. According to Table One in [64] the PAH contents are 8.8 mg/kg (Sterling V) and 0.039 mg/kg (Printex 90). Elder et al. [63] seemed to conclude that surface area plays an important role because Printex 90 (50 mg/m^3^) showed more pronounced responses than Sterling V (50 mg/m^3^) despite the 225 times higher PAH content of Sterling V. We emphasize that the low-surface area carbon black (Sterling V) was only applied in one single concentration in this inhalation rat study and that this concentration was very high (50 mg/m^3^). Thus, other mechanisms may be at work at such a high dosage because *“results of key studies reported for chronic inhalation of PSP [poorly soluble particles] in rats indicate that mechanisms of PSP-induced lung tumors at high doses do not operate at low dose levels”* [65]. Consistent with these results, a significant increase (P < 0.05) in 8-oxoGua (8-oxo-7,8-dihydro-2′-deoxiguanosine) induction was observed following 13 weeks of exposure to 50 mg/m^3^ Printex 90 and at 7 and 50 mg/m^3^ after the 44-week recovery period [66]. Interestingly, no increase in 8-oxoGua was observed for Sterling V at either time point. Although the retained mass dose of Sterling V at the end of exposure was even higher than for Printex 90 (50 mg/m^3^) (approximately 7.6 vs 4.8 mg), the surface area of the retained Sterling V was similar to that of the retained Printex 90 of the mid-dose exposure (7 mg/m^3^) (approximately 0.2 m^2^ in both groups). Sterling V (50 mg/m^3^) and Printex 90 (7 mg/m^3^) did not induce significant increases in 8-oxoGua in the lung at the end of the 13-week exposure. Gallagher et al. [66] noted that “*the lower effect per unit mass dose seen with Sterling V is consistent with earlier studies showing that particle surface area of low toxicity particles is a more appropriate dose metric for induction of inflammation in the lungs*”. We conclude that the observations in Elder et al. [63] are no reliable evidence against the importance of the surface area metric.

Finally, Tran et al. [67] developed an approach to estimate the no-observed adverse-effect levels in rats taking multiple lung compartments into account and using particle surface area as the relevant dose metric. They applied this approach to the data from Tran et al. [44]. This study derived a NOAEC of 3.5 mg/m^3^ for TiO_2_ and 7.5 mg/m^3^ for BaSO_4_, varying with different particle surface areas of the substances. In contrast to these findings, using a particle volume or particle mass based approach would return almost identical NOAECs for both substances.

As a side note, the corrected NOAEC estimate-based on the particle volume metric - was calculated at 7 mg/m^3^ for a substance density of 1 g/cm^3^ (see Table 4) which is in the range of the findings in Tran et al. [67]. Tran et al. [67] incorporated an additional “safety factor” of about 5 to address inter-animal variability (see Figure Seven in [67]) that almost compensates for the higher density of 4.25 g/cm^3^ (TiO_2_) and 4.5 g/cm^3^ (BaSO_4_). This crude coincidence in numbers should not be misinterpreted as a justification of the approach. Reliable translational toxicology models are difficult to develop and are too often believed to be reliable although having no validation [2,68].

The low toxicity of BaSO_4_ was also emphasized by Klein et al. [49]. Landsiedel et al. [53] reported on an inhalation study on rats exposed to 13 metal oxide nanomaterials and micron-scale zinc oxide for five consecutive days with 14- or 21-day post-exposure observation with concentrations ranging from 0.5 to 50 mg/m^3^. Bronchoalveolar lavage fluid (BALF) and histopathological sections of the entire respiratory tract were examined. Nano-BaSO_4_ did not induce any treatment-related effects up to an aerosol concentration of 50 mg/m^3^. These results are consistent with a previous study investigating BaSO_4_ effects upon intratracheal instillation [54], where bolus doses of 4.8 mg per rat lung did not affect any parameters of the BALF.

In summary, based on our review, retained surface area appears to be a reliable unifying denominator to assess pulmonary toxicity due to exposure to GBS. The most critical question to consider in using translational toxicology with any particulate substance however, is deciding on which of the many physico-chemical properties it may possess are most relevant (see Table Three in [69]). Thus, the weight of evidence indicates that no one metric can be applied to all GBS substances. In particular the findings with BaSO_4_, a GBS, challenge the basic assumptions of MAK’s translational toxicology models.

A recent report from ECHA outlining the best practices for human health and environmental risk characterization of nanomaterials came to very similar conclusion (see Section 3.2.1 in [70]). Thus, the approach of the MAK Commission which dismisses the particle surface area metric and does not test which metric is more appropriate under various circumstances appears unconvincing [11].

A search for the most appropriate effect metric (single or in combination) may be performed by multivariable statistical procedures successfully used in meta analyses. Unfortunately, these statistical tools are rarely used in toxicology although they can help to overcome drawbacks of elementary and univariable approaches applied currently [71-75].

### Epidemiology of GBS and related dusts

#### Coalmine dust: No lung overload and no lung cancer excess risk in workers

In an attempt to provide a perspective on risks of lung cancer under conditions of “lung overload”, we chose to review mortality studies of coal worker and other dust-related industry cohorts. Exposure to coal mine dust particulates in miners has long been recognized as one distinct occupation with significant potential for exposure to dusts, especially in past decades. It can be instructive to address the results of these studies in considering the potential human significance of high dose rat inhalation studies. Particle overload is typified by an impairment in alveolar particle clearance ([34], p. 1 and 4).

Intensive investigations in the US and in the UK showed that coalminers did not develop overload - even under high exposure conditions [33,76]. Kuempel et al. [33] studied pathologic data of 131 US coal miners (mean age at death: 67 years, average cumulative dust exposure: 107 mg-year/m^3^, 36 years of exposure, mean coal mine dust concentration: 3 mg/m^3^). The mean lung dust burden was 13.8 g (sd = 8 g) while the mean lymph dust burden, among the subset for which lymph data were available, was 1.6 g (sd = 1.6 g).

Tran and Buchanan [76] analyzed the pathological data of 423 UK miners: mean age at death: 67 years, average cumulative dust exposure: 256 gh/m^3^ = 145 mg-year/m^3^ (assuming 220 working days per year with a shift length of 8 h). The mean lung dust burden was 14.4 g (sd = 11.7 g) while the mean lymph dust burden, among the subset for which lymph data were available, was 2.3 g (sd = 1.0 g).

Kuempel et al. [33] referred to a dosimetric model developed in 1997 (PhD thesis of Eileen Kuempel) and found that a three-compartment model with no clearance breakdown fitted the lung burden best when analyzing the autopsy data of the US coalminers. Tran and Buchanan [76] tested this hypothesis in their independent and larger set of 423 UK miners and produced the same result. A best fit was achieved when the alveolar clearance rate was set invariant, i.e., the two independent studies present convincing evidence that even under the historically-high dust exposure scenarios of coalminers, no lung overload occurred in humans [33,76]. This result and the related Gregoratto model [32] were confirmed once more in a more recent study using both data sets in a Bayesian analysis via Markov Chain Monte Carlo simulations [77].

Coalminers do not suffer from elevated lung cancer risks [78,79]. In the most recent study on US coalminers [80] the lung cancer standardized mortality ratio (SMR) was only slightly elevated (SMR = 1.08, 95% CI: 1.00-1.18). This excess is unexceptionable because of the higher proportion of smokers at the start of the study in 1969/1970 (current smokers: 54%, Supplement Table Four in comparison to the US male population in 1970 (current smokers: 44.1%). Internal analyses showed an association of lung cancer mortality with coalmine dust exposure but only during the last follow-up interval from 2000 to 2007. All follow-up periods until 2000 showed no association between coal mine dust exposure and lung cancer [80,81]. The study relies on smoking information collected only at the start of follow-up. The models are unable to adjust for smoking habits after leaving work. Note that current smokers smoked less when working as a coalminer than current smokers in the US male population (prevalence of smoking more than 25 cigarettes per day: 12.4% among US coalminers vs. 28.0% in the US male population). This difference is probably caused by prohibition of smoking when working underground. It is plausible that smoking coalminers have increased their intensity of smoking after cessation of work underground and that this may have caused an increase in lung cancer mortality during the last follow-up period when most coalminers of the cohort have already stopped working underground (see the discussion of this issue in [82]). The US study [80] has an incomplete assessment of jobs held; no start and end date of jobs/tasks held before 1969/1971; no information on jobs/tasks held after start of follow-up in 1979/1971 and no end date of working as a coalminer for 16% of cohort members. Thus, only a crude assessment of exposure to coalmine dust up to the start of follow-up was possible: no time-dependent exposure analysis or lagging or lugging of exposures could be done. Crystalline silica concentration data suffered from additional limitations because measurements were available only after 1982 but had to be allocated to the jobs held before 1969/1971. Shortcomings and errors of this study were discussed in two Letters to the Editor [83,84]. The largest study to date with better assessment of exposures in a time-dependent manner was performed in the UK [82]: the overall evidence does not support an excess in lung cancer risk among coal miners, when compared to the general population or in internal analyses of the effect of coal mine dust exposure [85]. Similar results were found in Germany, based on a detailed and time-dependent exposure assessment in an analysis of lung cancer mortality and incidence data [79,86-88].

A study on US coalminer counties indicated cancer excess risks [89]. This study may be severely biased due to the ecological fallacy [90].

We would like to emphasize that all coalminer mortality studies discussed in this Section showed a link between coal mine dust exposure and coal worker’s pneumoconiosis, a clear sign of substantial dust exposure and tissue reaction. Thus, even in the presence of pulmonary fibrosis, no increase in lung cancer was reported in relation to coal mine dust.

#### Titanium dioxide, toner and carbon black: No lung cancer excess risk in workers

As with coal miners, no lung cancer excess risks were found in large cohorts of toner and TiO_2_-exposed workers as to be described below. A multi-center occupational epidemiology study was performed in Europe that enrolled 15,017 workers long-term exposed to TiO_2_ [91]. Four US production plants with a total of 4,241 exposed workers were studied [92]. An epidemiological investigation was performed on 33,671 workers with exposure to toner [93]. None found a lung cancer excess risk due to dust exposure. No evidence of adverse effects on pulmonary function indices and chest x-rays and no evidence of excessive inflammatory, allergic, or oxidative stress reaction was present in the toner-handling workers as compared to the nonspecifically exposed workers (1504 male workers in a Japanese toner and photocopier manufacturing company, means of personal 8 h respirable dust concentrations spanned from 0.012 mg/m^3^ in toner manufacturing to 0.989 mg/m^3^ in toner and photocopier recycling) [94-96]. The oxidative stress reaction was determined by urinary 8-oxoGua, a sensitive biomarker for increased repair of oxidatively damaged DNA. This biomarker has been successfully used in studies on environmental and occupational particle exposures (e.g., [97,98]). A Working Group at the International Agency of Research on Cancer (IARC) concluded that the evidence in humans for the carcinogenicity of TiO_2_ was *inadequate* [99,100].

Kuempel et al. [101] commented on a comparison of rat-based risk estimates (MLE, maximum likelihood estimates) by translational toxicology and epidemiological risk assessments: “*Regarding the magnitude of the excess risk estimates, the rat-based MLEs were clearly higher than the human-based estimate for coal dust (which was negative); however, the rat-based estimates (MLEs and 95% UCLs) did not exceed the 95% UCL from the human study (Table One). For carbon black, the rat-based excess risk estimates exceeded those from the human study, but the differences were not statistically significant. For titanium dioxide, the rat-based excess risk estimates (MLE and 95% UCL) were lower than the 95% UCL of the human studies, although the MLE from Fryzek et al.* [10] *was negative*”. These results of Kuempel et al. [101] showed that the rat findings are difficult to rely on when the toxicological effects of GBS dust in humans are to be estimated in quantitative terms. For coal mine dust and carbon black these authors found that the rat estimates are in excess in comparison to the humans. Because of statistical imprecision such a statement could not be derived for TiO_2_ but the authors stated that the epidemiological findings on TiO_2_ were negative.

The mortality of carbon black (CB) production workers has been extensively studied in the USA and in Europe [102-110]. Three major cohort epidemiological studies were performed in the UK, USA and Germany to investigate lung cancer mortality in CB production plants.

A UK cohort study on 1,147 workers at five plants [108] found a SMR of 1.73 (61 cases, 95% CI: 1.32 - 2.22). No trend across crudely assessed cumulative exposure, lagged up to 20 years was noted. Elevated lung cancer SMRs were observed at two plants: the SMRs of the other three plants were unexceptionable.

A German study of 1,528 workers at one plant [103,104,110,111] estimated an SMR = 1.83 (50 cases, 95%-CI: 1.34 - 2.39) but there was no link with CB exposures. However, the German study identified smoking and prior exposures to known carcinogens as important risk factors that could explain the major part of the excess risk [104]. A US cohort study on 5,011 workers at 18 plants [102] calculated an SMR = 0.85 (127 cases, 0.95-CI: 0.71, 1.00) and found no trend across time since first exposure and duration of exposure in years.

A Working Group at IARC concluded that the evidence in humans for the carcinogenicity of CB was *inadequate* [99,100]. An overview of these studies is described in McCunney et al. [112]: no lung cancer excess risk among CB workers could be established. This view is supported by studies in the CB user industries [113,114].

Since this IARC [100] evaluation, in an extended follow-up of the UK study, Sorahan and Harrington [109] applied a novel exposure metric (“lugging”) while hypothesizing that CB may act as a late stage lung carcinogen at plants with elevated SMRs. If so, the elevated SMRs of lung cancer should decrease substantially after cessation of exposure and positive associations should be found with “lugged” cumulative CB exposure (“lugging” the exposure by 15 years means to count only exposures received during the last 15 years). Sorahan and Harrington [109] observed both phenomena in those (and only those) two UK plant cohorts that had elevated lung cancer SMRs. The authors suggested that other investigators attempt to reproduce their findings. Morfeld and McCunney [105] tested this hypothesis in the German CB cohort. No decreasing SMR after cessation of exposure was observed, despite the fact that the German cohort showed an elevated lung cancer SMR.

Further analysis of the German CB cohort addressed potential “lugging” effects with a multi-model Cox regression approach [106]. This effort was designed primarily to explore the impact of cumulative exposure to CB “lugged” at 5, 10, 15, and 20 year - in other words, to evaluate the risk of lung cancer based on exposures within the most recent 5, 10, 15, and 20 years of exposure. Four cohorts within the overall cohort were evaluated including an inception cohort with different exposure scenarios. Despite extensive searching, 719 models returned negative coefficients. Only one model estimated a small positive, nonsignificant coefficient. This analysis did not support the hypothesis of CB being a late-stage carcinogen [109].

Bayesian analyses were also employed [107] to explore potential risk factors and confounders that may have contributed to the SMR lung cancer results. When putting a flat prior to the SMR a Markov chain of length 1,000,000 returned a median posterior SMR estimate (that is, the adjusted SMR) in the range between 1.32 (95% posterior interval: 0.7, 2.1) and 1.00 (0.2, 3.3) depending on the method of assessing previous exposures. These additional studies provide further support for the lack of an increased risk of lung cancer as a result of working in the CB – producing industry.

A US cohort study on 5,011 workers at 18 CB – producing plants [102] calculated an SMR for lung cancer of 0.85 (127 cases, 0.95 CI: 0.71, 1.00) and found no trend across time since first exposure and duration of exposure. No estimates of exposure intensities or cumulative exposures were available for this cohort.

The relationship between workplace exposure to CB and lung cancer risk was examined in two large population-based case–control studies carried out in Montreal, Canada Study I: [113], Study II: [114]. Interviews for Study I were conducted in 1979–1986 (857 cases, 533 population controls, 1,349 cancer controls) and interviews for Study II were conducted in 1996–2001 (1,236 cases and 1,512 controls). Detailed lifetime job histories were elicited and a team of hygienists and chemists evaluated the evidence of exposure to a host of occupational substances, including CB. Lung cancer risk was analyzed in relation to each exposure, adjusting for several potential confounders, including smoking. Subjects with occupational exposure to CB, TiO_2_, industrial talc and cosmetic talc did not experience any detectable excess risk of lung cancer.

In summary, no causative link between exposure to well-investigated respirable GBS (including some nano-structured dusts) such as coal mine dust, TiO_2_, toner or CB and no excess in lung cancer risk in humans has been demonstrated.

### BAL studies in humans are consistent with epidemiological results

As discussed before, epidemiological data do not provide convincing support for an increased lung cancer risk in people exposed to high dust levels, such as coal miners. Epidemiological findings contrast with the results of experimental studies on rats, in which at higher exposure levels, excess lung tumours were detected. Chronic inflammation is the underlying mechanism, which causes secondary genotoxic events by oxidative damage due to inflammatory cells. Consequently, prevention of inflammation is the rationale for the establishment of threshold values (MAK values) by the MAK Commission [11]. The experimental studies were evaluated mainly by analyses of inflammatory cells (PMNs) in the BALF (e.g. [14]). Thus the BALF-PMN in the rat experimentation operates in a dual way: (a) the “causative (true)” biomarker for the prevention goal and (b) the parameter for the assessment of the NOAEL and the derived setting of MAK values.

BAL is a widely used clinical diagnostic study in the evaluation of lung disorders, particularly in the differentiation of interstitial lung diseases (ILD). In light of the emphasis given by the MAK Commission to data from rat experiments, it would be valuable to determine whether corresponding biomarkers can be identified in human BALFs of dust-exposed people. BALFs on coal workers were assessed for their cellular profile [115-119]. Groups of miners with different stages of coalworkers’ pneumoconiosis (CWP) were compared (posterior-anterior chest radiographs, ILO resp. Chinese x-ray staging of CWP). No increased counts for PMNs were detected in asymptomatic miners [115-117] and in miners with low grades of simple pneumoconiosis, i.e., CWP ≤ 1/1 [115,116]. One group of miners with simple pneumoconiosis showed an elevation of the neutrophil percentages in the BALF in comparison to controls [118]. In contrast, a second group studied by the same researchers showed almost the same average neutrophil percentage as reported for controls [119]. Unfortunately, the distribution of CWP degrees was not given in these studies and the potential overlap of both investigations is unclear. We note that the recovery techniques may have differed between research groups and the frequency of neutrophils were reported on varying scales (percentages, counts per ml).

Xing et al. studied biomarkers in the BALFs of coal mine workers: 14 active underground miners without CWP, 21 workers with CWP 0/1, and 13 no longer exposed workers after cessation of exposure with CWP 1/1. None of the groups showed elevated neutrophils numbers (PMNs). However, other biomarkers in the BALF of the coal workers were clearly changed; for instance markers of the epithelial reaction (pneumocyte type II): (a) increased surfactant lipids, (b) altered ratio of PG/PI (subgroups of lung surfactant: phosphatidylglycerol PG, phosphatidylinitisol PI), (c) increased surfactant protein A. The elevated TNF alpha content in the BALF (d) stands for the effect of the phagocytosed particles on AM. Interestingly, the results on parameters (a, b, d) correspond to findings in dust-exposed rats, e.g., the increased surfactant lipids, the altered ratio of PG/PI, the elevation of TNF alpha [120-123]. It is worth mentioning that rats exposed to coal dust showed a significant increase of PMNs in the BALF, e.g. [124]. The investigations of Vanhee et al. [125] identified different profiles of growth factors (PDGF, IGF1, TGF beta) in the BALF of coal miners according to the severity of x-ray changes. Further in vitro and in vivo studies on human (BALF-) alveolar macrophages from patients with different grades of pneumoconiosis clearly demonstrated the eminent role of the AM for the onset and development of the coal miners’ lung disease [119]. Mixed CS and coal dust exposures eventually trigger an aggressive form of pneumoconiosis and BALF pattern [117]. The miners’ individual working-lifetime exposures (n = 20) were estimated from this study, using work histories and airborne mine dust data. The quartz lung-burdens were calculated using a lung dosimetry model. The study showed that quartz, either as cumulative exposure or as calculated lung burden, was a highly significant predictor of PMN lung response. The cumulative coal dust exposure did not contribute to the prediction of PMNs [126].

An ATS clinical practice guideline on the utility of BALF cellular analysis [127] summarized for CWP that BALF cell profiles, indicative of increased numbers of macrophages and elevated proportion of coal dust-laden macrophages, are suggestive of CWP or progressive massive fibrosis (PMF). The authors stated for silicosis that BALF profiles of silica-exposed workers and workers with silicosis are characterized by an excess in BALF macrophages and an increased silica particle burden of macrophages that is appreciable in non-smokers. Meyer et al. [127] made no recommendations regarding the clinical utility for prognosis of CWP or PMF. The authors noted about the prognostic value for silicosis that increased numbers of lymphocytes and neutrophils have been associated with progression to silicosis.

In conclusion, the prominent role given to the BALF-PMNs in relation to the particle lung exposure in rats does not correspond to BAL results in humans. Human data reflect a significant role for the alveolar macrophages [128] and type II pneumocytes in the development of dust induced ILDs in humans, a role also played in rat studies. The PMNs, however, play a unique role in rat experiments, findings that do not appear to occur in high dust exposed workers, such as coal miners. In conclusion, the human BAL biomarker studies corroborate the epidemiological findings described in the earlier Section.

### Comparative interspecies responses to GBS exposure

#### Species-specific response to GBS in inhalation studies

Although the rat has been the experimental species most extensively used in GBS investigations and there is thus, an abundance of pathophysiological and toxicokinetic data for a range of GBS, it is important to consider how relevant all this information is when reading across, using translational toxicology to other species and in particular, humans. In a Letter to the Editor Kuempel et al. [10] noted that there are similarities between the human lung responses to respirable particles compared to those observed in rats exposed to overload doses. The authors stated that an “*ILSI expert panel concluded that the rat is a useful model for non-neoplastic lung responses to poorly-soluble particles and that (in the absence of mechanistic data to the contrary) it is also relevant to identifying potential carcinogenic hazards in humans.*” This contrasts somewhat with a description of the ILSI panel [129] given on page 4 in [34]:

“*The main conclusions from this ILSI workshop on ‘lung overload’ can be summarised as follows:*

- *Hallmark of particle overload is impaired alveolar clearance.*
- *Precise mechanisms are not known but volumetric inhibition of macrophages and the development of an inflammatory environment seem to be important drivers.*
- *Differences in potency of various PSPs are obvious and are leading to the need of dosimetric adjustments accounting for differences in deposition and clearance of particles.*
- *Overload is not a rat specific phenomenon and seems to be generally reversible but may reach conditions where clearance impairment is irreversible.*
- *Overload contributes to the (species independent) pathogenesis of non-neoplastic lung responses and is a prerequisite for the tumorigenic effects observed in rats. With regard to humans, despite evidence that particle clearance is impaired in many coal workers, no conclusive evidence for increased lung cancer risk exist for workers chronically exposed to coal dust or for workers exposed to other poorly soluble particles.*
- *For neoplastic lesions, dose–response data from persistent neutrophilic inflammation and cell proliferation can be used as surrogate for risk characterization.*
- *For non-neoplastic responses, persistent neutrophilic inflammation may also be used a surrogate whereas epithelial cell proliferation is not considered a necessary prerequisite for fibrosis.*
- *A nonlinear dose–response approach for the characterization and evaluation of both, neoplastic and non-neoplastic lesions are considered plausible based on the assumed pathogenesis.*
- *An uncertainty factor of 1 for both neoplastic and non-neoplastic endpoints can be considered sufficient to account for toxicokinetic and toxicodynamic parameters.*
- *With regard to an appropriate dose metric some estimate/parameter reflecting retained lung burden is recommended together with a full characterization of the aerosol exposure parameters (e.g. MMAD, particle surface area, density).*
- *With regard to non-neoplastic responses the rat is considered predictive of a non-neoplastic hazard for humans.*
- *With regard to neoplastic responses the rat is considered to be more responsive than other species including humans at doses and exposure intervals that result in pulmonary particle overload.*
- *The mode of action for induced neoplastic responses in rats apparently needs accumulation of particles in lung alveolar and interstitial compartments, persistent inflammation and epithelial cell proliferation*”.

We thus note that there is a continuing debate regarding the similarities (nature and extent) of the effects in rats (and other experimental species) and humans. In the following, we will review species differences again, but focusing on the MAK Commission’s approach. In the translational Model B used by the MAK Commission, much is predicated on the rat lung alveolar macrophage (AM) responses to GBS. However, it is well know that there are important differences in species differences in composition, localization, and function between the different AM subsets which may well account for some of the observed differences in responses to inhaled GBS.

As one example, this is demonstrated with the finding that the shortest AM clearance times are reported for rodents where deposited particles remain on the epithelial surface of the lung [130,131] whereas, longer clearance half times are found in humans, monkeys, dogs and guinea pigs [132,133]. Species differences exist also in the cell size of AMs, with those from humans being significantly larger than those from rats, hamster or monkeys. These differences in size have been considered in the AM pool volume model [14] and in Model B of the MAK Commission [11]. In addition, it has been observed that both the number and size range of phagocytised particles vary among species [134].

To highlight this difference in AMs between species, it is interesting to note that Dörger et al. [135] and Jesch et al. [136] reported that nitric oxide formation was only observed by rat AMs, but not in the AMs from hamsters, monkeys or humans. The authors concluded that specific regulatory mechanisms of the nitric oxide pathway in AMs from these four different species existed.

In spite of extensive research, it still remains unclear why rats alone respond with the development of lung tumours, but other animal species, chronically exposed to GBS, do not. Clearly, the role of stimulated AMs and PMNs is important as lung tumours have never been reported in rats when pulmonary inflammation was absent [129,137-140].

The greater sensitivity of the rat lung with regard to oxidative stress and subsequent epithelial cell responses is most likely due to a more pro-inflammatory environment compared to other experimental species. The consistent finding that lung tumours in rats following chronic exposure to GBS are induced by such an indirect mechanism is supported by results of other experiments. Inflammatory cells and activated AMs, which are found in large numbers in animals exposed to GBS, can release ROS and other mediators of inflammation, which in turn, are able to induce DNA damage by a secondary mechanism [138,141-143]. In contrast to this finding in rats, no other animal species, including mice and hamsters have been reported to have developed lung tumours following such chronic exposure to GBS.

Of particular importance for risk assessment is the observation that the pulmonary responses of rats are extremely marked when compared to other large mammalian species such as non-human primates and humans. It has been proposed that the intrapulmonary particle retention patterns and tissue reactions in rats may not be predictive of pulmonary retention patterns and tissue responses in either primates or humans as reported by Nikula and coworkers [144,145]. In these studies, male monkeys and rats were exposed for 7 hours/day, 5 days/week for 24 months to diesel exhaust particulates (2 mg/m^3^), coal dust (2 mg/m^3^), or diesel exhaust particulate and coal dust combined (1 mg/m^3^ each) and were subsequently examined histopathologically. In all the exposed groups, monkeys retained a similar amount or more particulate material in the lungs than did the comparative rats groups. Exposed rats retained a greater fraction of the particulate material in the alveolar ducts and alveoli, whereas monkeys retained a greater proportion of particulate material in the interstitium. Most importantly, rats, but not the monkeys, developed significant alveolar epithelial hyperplastic, inflammatory, and septal fibrotic responses to the retained particles. It was proposed by the authors that these differences in particulate tissue distribution in rats and humans might bring different lung cells into contact with retained particulates or particle-containing macrophages. This may, in part, account for the differences in species responses to inhaled GBS.

The authors concluded: *“These results suggest that intrapulmonary particle retention patterns and tissue reactions in rats may not be predictive of retention patterns and tissue responses in primates exposed to poorly soluble particles at concentrations representing high occupational exposures. The pulmonary responses of the rats were severe compared to the primate, where the insult to the lungs was handled without adverse consequences”* [144].

Nikula et al. [35] also have demonstrated that the relative amounts of intraluminal and interstitial particle load differ markedly between rats and humans with particles being found predominantly in the interstitium in man and intraluminarly in rats. This is consistent with the finding that acute intra-alveolar inflammatory responses, alveolar epithelial hyperplasia and alveolar lipoproteinosis were all significantly more pronounced in rats compared to humans exposed to the same particles [146]. This further supports the author’s contention above that these differences may also account for the species differences seen in the long-term responses to high GBS exposures.

The IARC Working Group [147] noted that the dose metric that best describes the dose–response relationship for GBS with lung tumour induction in the rats can be surface area, particle and size [148-150]. Interestingly, they remark that the degree of sustained inflammation experienced by rodents (most notably rats) at high lung burdens has not been observed in humans. It is of particular relevance to note their following conclusion in regards to interspecies responses to GBS.

*“Rats and mice, in contrast to hamsters, exhibit sustained inflammation associated with particle lung burden, but lung tumours induced by poorly soluble particles have only been observed in rats. It has been shown that rats are uniquely susceptible to poorly soluble particle-induced lung cancer relative to mice and hamsters. While some of the steps indicated in Figure 4.2 have been demonstrated in humans exposed to poorly soluble particles, it is not known to what extent humans are susceptible to particle-induced lung cancers associated with titanium dioxide, carbon black or talc”* [147].

Another approach to considering the interspecies lung reaction to GBS is the use of the “Adverse Outcome Pathway” (AOP) approach. This model describes the sequential progression of events evolving in an organism from the first contact of a toxicant at the molecular level, via a subset of following key effects or biological responses to a final adverse outcome at the individual or population level [151]. Although AOPs can be outlined as a linear cascade of consecutive events, where one common molecular initiating effect is the prerequisite for all subsequent steps, the “adverse outcome” may vary significantly. In this respect, AOPs take into account that different molecular initiating events can cause the same adverse outcome as well as that many different “mode-of actions” (MoA) share common key molecular initiating events. Even though the adverse outcome observed *in vivo* is the result of a sequential cascade of biological events, each step in this pathway may itself be influenced by other pathways ongoing and/or dominating within the biological system of interest.

This AOP approach has been recently used to describe an interspecies comparison of response to high exposures to GBS [34] in experimental rodent species and humans. Although an accumulation of particles in the lung is a common finding in all investigated species, significant differences in the phenotypic “adverse outcome” between rats and all other mammalian species, including humans, exist. As noted earlier, lung tumours have been reported exclusively in rats, but not in mice, hamsters, non-human primates or humans. It is well established that lung “overload” also contributes to the observed (species independent) pathogenesis of non-neoplastic lung responses, with the significant impairment of pulmonary particle clearance as “initial event” relevant for AOP considerations.

The application of the AOP approach to a number of chronic inhalation bioassays with a range of GBS and other experimental data leads to a helpful summary of findings as exemplified with Table 5 taken from ECETOC [34].

#### Species-specific response to GBS on the cellular and molecular level

One can explore the AOP approach by examining toxicological studies that have investigated species-specific differences with regard to the species-specific responses to GBS on the cellular and molecular level, a condition which makes up a central hypothesis in the MAK Commissions’ GBS document [11]: Basic assumptions are that secondary genotoxic mechanisms underpin particle genotoxicity and tumourgenicity. *In vitro* and *in vivo* toxicological studies have consistently demonstrated that the tumour induction in lungs of rats by particles is closely linked to inflammation and ROS released by excessively particle loaded alveolar macrophages and by secondary elicited PMN [148,152,153]. The critical events are listed in Table 6 below.

ROS is highly DNA reactive and leads to mutagenic DNA modification such as 8-oxoGua [121,154]. Normally, cells possess potent defense mechanisms leading to a steady state level of 8-oxoGua via antioxidants systems as GSH and SOD. Inflammation evokes higher amounts of ROS, eventually overloading the defense mechanisms. However, adaptive responses compromise higher expression of higher antioxidant molecules (front line defense), which according to the experimental data is also species specific (see Table 7). In proliferative competent cells, a DNA damage check point arrests cell cycle via cell autonomous responses to allow time for any DNA damage to be repaired [155] leading to genomic maintenance. In the case of a severely DNA damaged cell, the DNA checkpoint advances tumour suppressor mechanisms such as apoptosis or senescence.

Basically, the GBS - alveolar macrophage interaction initiates a cascade of events, which eventually leads to critical biomarkers of mutagenic oxidative DNA damage (8-oxoGua) (see scheme in Table 6). To explore the AOP approach, data from three studies were extracted: A subchronic inhalation study with CB which compares the key pro- and anti-inflammatory markers of rat, mice and hamsters [156]. These markers were allocated to the phase 1 and 2 levels of particle lung interaction (see Table 6). The two other studies considered, take into account the lung reaction after crystalline silica (CS) exposure of rat and hamsters.

CS has a high surface activity which stimulates inflammatory responses in the lungs of rodents. After CS particle exposure the rat model presents the same essential cellular and molecular events (Table 6), which are relevant for the GBS inflammatory potential at significant higher doses [121]. This cascade of events following exposure to CS particles includes [123]: ROS release from AM, elicitation of inflammatory mediators, recruitment of PMN, radical scavenging, mutagenicity, oxidative DNA damage. Taken together, critical steps in the leading section of the AOP to tumour formation in the rat model are identical after (high) GBS and (low) CS exposure. Thus, an approach to compare the different steps (see Table 7) in different species (rat, mouse hamster) including data from CS studies sounds reasonable.

One acute study tested CS (Min-U-Sil) at two moderate doses (0.2 and 2 mg) and one high dose (30 mg) via intratracheal installation on rats and hamsters (160–180 g BW) and a post exposure interval of 7 days [157].

Both species were studied for lung reactions via BALF cell count (PMN), BALF cell oxidant and NO production and, expression of pro-inflammatory and anti-inflammatory mediators. Both species responded to the CS challenge (hamster essentially to the extreme dose, 30 mg). However rat showed much higher reactions in this acute study than hamster in all parameters investigated.

The second CS study analyzed the lung reactions of rat and hamsters at two moderate doses of quartz

**Table 5:** **Interspecies lung responses** ^**a**^ **following long-term or chronic inhalation exposure to GBS**

**Species**			
**Rat**	**Mouse**	**Hamster**	**Primate/human**
Likelihood for developing particle overload (slow lung clearance)			
+++	+++	+	Not determined*
Alveolar macrophage participation			
Active (accumulation in alveolar ducts)	Active (accumulation in alveolar ducts)	Extensive (rapid clearance)	Not as extensive (translocation to interstitial sites)
Pulmonary (neutrophilic) inflammation			
+++	+++	+	+
Epithelial and interstitial cell proliferation			
+++	+	(+)	(+)
Septal fibrosis			
+++	+	(+)	(+)
Anatomical location of retained particulates			
Primarily alveolar (some increased translocation at overload)	Primarily alveolar (some translocation at overload)	Rapid clearance	Primarily interstitial
Lung tumours following chronic exposure			
Yes	No	No	No

^a^Severity low +, moderate ++, high +++, or questionable (+), reprinted with permission from ([34], p. 52)**.

*This should be + (see p. 53 in [34]) because particle overload is typified by an impairment in alveolar particle clearance (see p. 1 and 4 in [34]).

**There may be a variance of opinion about the extent/degree of some of the endpoints in the table (e.g., alveolar macrophage participation, septal fibrosis) and there is continuing research to refine these findings.

**Table 6:** **Cascade of cellular and molecular biological events following particle lung exposure**

**Phases**		**Hallmark**
Phase 1	Production of inflammation promoting mediators	● Stimulation of primary ROS from AM, RNS
		● Stimulation of secondary ROS, RNS from AM, PMN (epithelial cells)
		● TNF alpha, MIP2 from AM
		● PMN recruitement
Phase 2	Increased production of anti-inflammatory mediators	● GSH
		● SOD
		● Anti-inflammatory cytokine IL-10
Phase 3	Repair of injury	● Stimulation of DNA-repair mechanisms
Phase 4	Intermediate endpoints	● 8-oxoGua
		● Proliferation

Abbr.: 8-oxo-7,8-dihydro-2´-deoxiguanosine: 8-oxoGua, alveolar macrophages: AM, reactive oxygen species: ROS, reactive nitrogen species: RNS, glutathione: GSH, macrophage inflammatory protein 2: MIP 2, polymorphonuclear neutrophils: PMN, superoxide dismutase: SOD, tumour necrosis factor alpha: TNF alpha.

**Table 7:** **Comparison of initial cellular and molecular events after lung particle exposure in different experimental animals leading to pre-tumour conditions: DNA damage, p53 activation and proliferation**

**Phase #**	**Parameter**	**Animals compared; model used**	**comparison**	**Source**
Phase 1	PMN	BALF; rat mouse hamster	R >> > M, H	Carter and Driscoll [156]
				Carter et al. [157]
Phase 1	PMN	BALF; rat, hamster	R >> > H	Seiler et al. [158]
Phase 1	MIP2, TNF alpha	BALF AM; rat mouse, hamster	R >> > H, M	Carter and Driscoll [156]
				Carter et al. [157]
Phase 2	IL-10	Rat, mouse hamster	R, M < << H	Carter et al. [157]
Phase 2	GSH level in BALF	Rat vs. hamster	R >> > H	Seiler et al. [158] (it 90d)
Phase 3	Repair: Indirect hint by in vitro studies of the ionizing radiation induced DNA damage (Human vs murine cells),with regard to the species-specific reaction to particles differences in repair capacities are not investigated			Behrens et al. [159]
Phase 4	8-oxogua	Tissue; rat vs. hamster	R >> > H	Seiler et al. [158]
Phase 4	P53 Mutation in tissue	Tissue; rat vs. hamster	R > H	Seiler et al. [158]
Phase 4	proliferation	Tissue; rat vs. hamster	R >> > H	Seiler et al. [158]
				Carter and Driscoll [156]

Abbr.: rat, R; mouse, M; hamster, H; reduced glutathione, GSH; superoxide dismutase, SOD.

(DQ12) (0.3 and 1.2 mg/10 g bw) in a 90 day sub-chronic assay [158]. Phase 1 and 2 parameters (see Table 6) were similar to the acute study on CS reported above: rats showed stronger lung reactions with regards to the inflammatory biomarkers than hamster. Persistent elevated levels of 8-oxoGua in rat cells but not in hamster cells *in vivo* demonstrate significant differences in the development of persistent mutagenic oxidative damage and proliferation and may explain the different outcomes in rat and hamsters with respect to tumour development (see Table 7). Importantly, both CS studies found no proliferative response in hamster lungs after CS exposure; thus contrasting the strong and dose-dependent proliferative reaction in rat lungs (phase 4 effect).

The inclusion of phase 4 effects in comparing rat vs. hamster assesses the real “point of no return reaction level” in the cascade. The level of persistent oxidative DNA damage, in conjunction with a continuing proliferative stimulus, appears to constitute a prerequisite condition for tumour development via this secondary genotoxic mechanism. In support and amplification of the “Adverse Outcome Pathway” (AOP) approach described in the Section on species-specific response above, the data of phase 3 and 4 provide relevant information pointing to the final adverse outcome [160].

#### Species-specific response to GBS: Conclusions

As can be seen from the above discussions, it is possible to explore the species differences using the MoA, the AOP and events at the molecular level to help us better refine the way we use translational toxicology to exchange experimental findings between rodent species, primates and human responses to GBS. From the wealth of available data, it seems too simplistic to simply assume that what occurs in the rats can be assumed to occur in humans without carefully taking into account both critical toxicokinetic and toxicodynamic differences. This means that we have to take into account the totality of the available information at the anatomical, physiological, cellular and molecular level in a reliable translational exercise. Rats have been consistently shown to have a more sensitive response to the chronic inhalation of respirable particles compared to other species, and a unique response in relation to lung cancer. The species-specific differences in responses are summarized in Table 5. Thus, in agreement with ECETOC [34] we conclude that mechanistic data are available to overcome the default statement made by the ILSI panel in 2000 [129] and cited in [10]. This conclusion is consistent with findings from studies on humans (see sections on epidemiology and BAL studies above).

The basic assumption applied in both Model A and B to translate rat findings to humans is one of a “species independent” effect of GBS when expressed using specific metric scales. However, variable responses, at the cellular and molecular levels, as well as regarding tumour development (defense systems) are seen in mice, hamster, rats, and primates following particle exposure. It is thus important to ascertain how these models perform in a translational exercise between these three and possibly other species in order to verify the “species independent” assumption. Such a validation exercise should be performed prior to their use in deriving exposure limit values for humans [161].

## Some comments on measured occupational exposures levels

The MAK Commission [11] derived a respirable concentration limit (OEL) for GBS. The MAK Commission made extensive use of toxicological results on CB and TiO_2_. Both are leading examples of substances investigated repeatedly in studies on dust effects. In the following we present and discuss occupational exposure levels of CB and pigmentary TiO_2_ in order to provide an overview of current or past exposure conditions at the work place.

**Figure 1:**
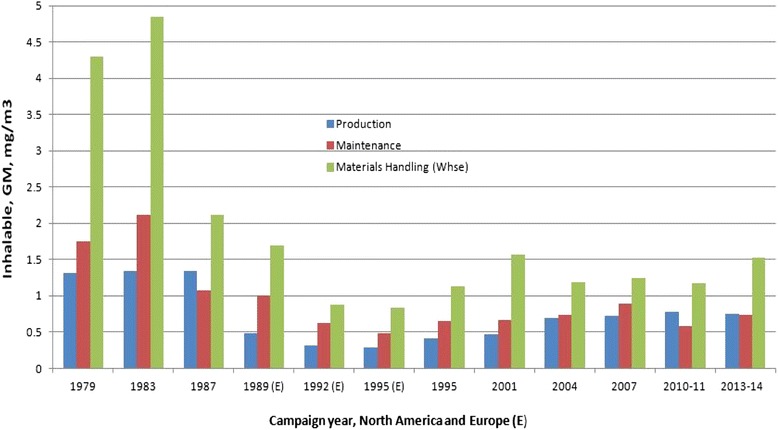
**Inhalable carbon black concentration, geometric mean (GM) exposures for production, maintenance, and materials handling (warehousing), highest exposure job classes in carbon black manufacturing operations, 1979–2014.**

The CB producing industry has conducted industry-wide exposure assessments at approximately 40 CB manufacturing plants in North America and Europe in support of epidemiology studies, internal and external occupational exposure level (OEL) development, and other industrial hygiene applications. Between 1979 and 2014 more than 13,500 inhalable TWA personal samples have been collected, and in the period up to 2001 nearly 9,400 respirable TWA personal dust samples were collected. These samples documented worker exposures by major job class and job title [162-172]. Figure 1 presents inhalable dust exposure trends over the past 35 years for three of the major job classes associated with CB production in Europe and North America. Two comprehensive respiratory morbidity studies were completed in Europe and North America in the mid-1990s and early 2000s, respectively [163-168,171,172]. One of the outcomes of these studies was that the inhalable dust fraction was affirmed as the most appropriate metric for assessing health risks in the CB producing industry based on reported findings of bronchitis and small, but statistically significant, decrements in one aspect of lung function over a 40 year period.

In 2010 the ACGIH® Threshold Limit Value (TLV®) Committee adopted a revised TLV for CB of 3.0 mg/m^3^ TWA, inhalable (<100 μm, aerodynamic diameter) [173]. This was the first revision to the CB TLV since its original adoption in 1967. The ACGIH also revised its 1996 cancer classification for CB from A4, Not *Classifiable as a Human Carcinogen, to A3, Confirmed Animal Carcinogen with Unknown Relevance to Humans.* While the TLV Committee considered animal toxicity studies related to CB in its deliberations it also fully evaluated the extensive worker-based epidemiology studies reported in the peer reviewed literature [173]. Following a thorough review of all relevant animal and human health effects information, the ACGIH TLV Committee independently identified the inhalable dust fraction as the most appropriate metric for a health-based OEL for CB [173].

**Figure 2:**
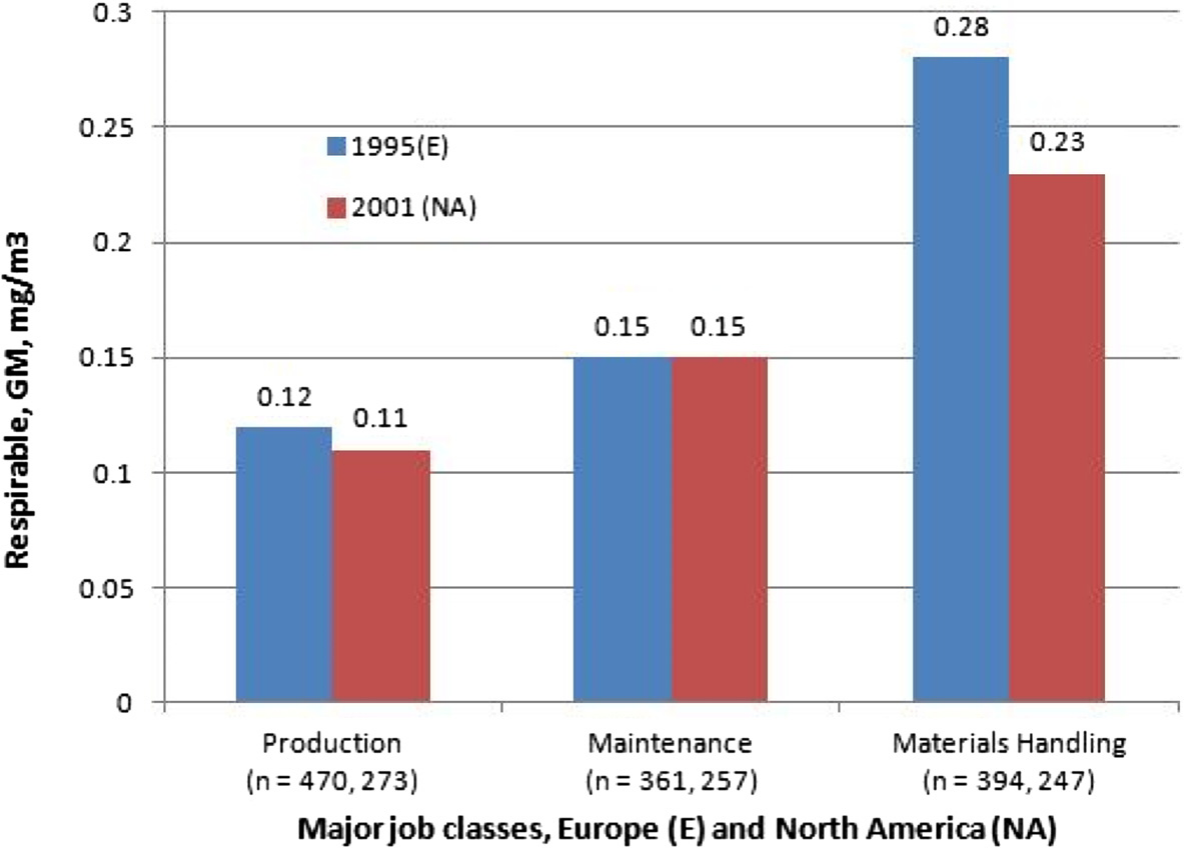
**Respirable carbon black concentration, geometric mean (GM) exposures for production, maintenance, and materials handling (warehousing), highest exposure job classes in carbon black manufacturing operations in most recent respirable dust study years.**

In light of the outcomes of European and North American respiratory morbidity studies and the inhalable metric that the TLV Committee was in the process of adopting for CB, a low solubility low toxicity particle, the CB producing industry ceased measurements of the respirable dust fraction in 2001. Figure 2 presents a summary of respirable dust concentrations for the last industry-wide exposure measurements conducted in Europe and North America in 1995 and 2001, respectively.

It should be noted that nanostructured aggregates and/or agglomerates have been the relevant subset of working lifetime exposures to dusts, such as they may have existed, within the worker populations of CB manufacturers for more than 140 years. To better quantify the nature of small particle exposures in the industry a comprehensive particle characterization study at several CB operations was conducted in 1999. The study concluded that airborne CB particles associated with bag filling had a size distribution starting at approximately 400 nm and that CB ultrafine particles (<100 nm, aerodynamic diameter) did not exceed background levels around reactors and pelletizing operations under normal operating conditions [174,175]. A survey of ultrafine aerosols in various UK industries, that included a CB producing plant, did not measure ultrafines above ambient background in the bagging operation [176].

Large hygiene and epidemiological studies were performed on workers in the US and European pigmentary TiO_2_ production industry (USA [92,177], Europe [91,178]). An overview was given by Hext et al. [56]. The European multicenter study comprised 27,522 workers from 11 plants from six countries. Exposure reconstruction was based on personal dust measurements mainly performed during the 1990s. Average respirable dust concentrations of TiO_2_ dropped from about 0.3 mg/m^3^ to 0.7 mg/m^3^ in the 1950s to current typical levels of about 0.2 mg/m^3^ to 0.3 mg/m^3^. The maximal yearly averages were reported in some jobs as 8 mg/m^3^. Inhalable dust concentrations were estimated to be higher by a factor of 3.3 on the average, with a maximum at 26 mg/m^3^ [178]. The US study included 4,241 workers from four production plants. In contrast to the European study, only the long-term area samples were used. The median values fell from 4.6 mg/m^3^ between 1976 and 1980 to 1.1 mg/m^3^ between 1996 and 2000. Packing, micronizing or internal recycle workers showed a median exposure at 3.0 mg/m^3^ in comparison to median levels of 0.3 mg/m^3^ and 0.9 mg/m^3^ for other jobs.

As reported in the Section on epidemiology above, no cancer excess risk has been found under these exposure conditions. It appears that the use of epidemiological evidence should be considered in the derivation of occupational exposure limits like those of GBS. This may also help to define the most relevant dust metric for the measurement of work environment exposures.

## Discussion

The calculations described in the MAK document [11] on GBS are based on a number of incorrect assumptions and calculations related to the use of lung surface area, particle clearance rates and deposition fractions among others which are shortcomings that affect both translational overload models (Model A and Model B) used to derive the HEC for GBS. The methods applied do not reflect state of the art techniques and cannot be independently replicated since the hyper link cited by the MAK Commission [11] no longer leads to the program version the Commission and Pauluhn [14] applied (MPPD 2.0). In [14] calculations were based on a Fortran program that is not publicly available. More importantly, the approaches are inconsistent as they rely on conflicting assumptions. The resulting errors are so large that the MAK Commission’s suggestion [11] as to how to translate inflammation/overload findings from rats to humans is unreliable and the OEL proposal is unsubstantiated. This also affects the justification of the MAK Commission’s cancer classification [11] which is related to humans (Carcinogen Category 4) but based on overload inhalation experiments with rats. This classification relied on the validity of the proposed translational overload models.

The effect metrics selected by the MAK Commission [11] and used in Pauluhn [14] did not consider particle surface area despite toxicological evidence in favour of this metric. No quantitative analysis was presented that could justify why the role of particle surface area was ignored.

The MAK Commission [11] did not discuss that workers exposed to high dust levels (coalminers) showed no evidence of dust overload, demonstrated no lung cancer excess risks and that BAL findings in humans did not replicate the PMN elevations seen in rats, even though signs of dust effects in the BALF (change of surfactant lipids, and SP-A, TNF alpha increase) as well as pneumoconiotic pathology were registered. In addition, epidemiological studies on CB and TiO_2_ exposed workers did not find a lung cancer risk that could be related to the workplace exposures. Thus, there was no evidence presented and there is no evidence available that overload findings in rats have relevance to humans in cancer classification or limit value assessment applying the translational models used by the MAK Commission [11]. In an extensive epidemiological application lung burden models did not fit any better than typical cumulative exposure models: correlations were always higher than 0.95 [179].

The MAK Commission [11] did not test whether the suggested overload models reliably “translate” rat findings to other species, like mice and hamsters. Because mice and hamsters react differently to GBS than rats, we believe that an attempt to validate the models across these three animal species (and others) is necessary before any application to humans should be suggested in limit value assessment [161]. We emphasize that the endpoint chosen by the MAK Commission (markers of inflammation and overload in the lung lavage) is invasive and cannot be used in occupational medicine programs. Peripheral markers should be developed and tested in humans before these endpoints investigated in rats are considered to be relevant for monitoring workers for early signs of pulmonary inflammation.

We explored species-species differences and concluded that it seems too simplistic to assume that what occurs in the rats occurs in humans after adjusting for some anatomical and physiological differences. Significant differences in the adverse outcome pathway (AOP), at the cellular and molecular levels, as well as regarding tumour development (defense systems) are seen in mice, hamster, rats, and primates following particle exposure.

Reliable translational toxicology models are difficult to develop and are too often believed to be reliable although having no validation [2,68,180]. Pound et al. [68] concluded that *“the value of animal research into potential human treatments needs urgent rigorous evaluation”* and Seok et al. [2] found that *“genomic responses in mouse models poorly mimic human inflammatory diseases”*. So it is not surprising that our critical evaluation of the suggested translational overload models provided no support to the MAK Commission’s proposal for GBS [11]. The new approach suggested does not meet general criteria of scientific reliability: it is non-transparent, it is inconsistent and it is not evidence-based. Moreover, it fails in a critical area of science: using the methods proposed, the results could not be reproduced.

We believe that a balanced scientific derivation of limit value proposals for GBS and cancer classification should use all information available and also rely on epidemiological studies. This was so with the former approach chosen by the MAK Commission [15]. They derived an MAK value for the respirable fraction of 1.5 mg/m^3^ and 4 mg/m^3^ inhalable. The new approach [11] is based on translational toxicology models exclusively and ignores epidemiological evidence. A derivation based on epidemiological findings is suggested by other authors and institutions who argue that a 1 mg/m^3^ respirable limit may be used as a starting point for detailed discussions [9]. The Institute of Occupational Medicine (IOM) has recommended an exposure level of 1 mg/m^3^ for respirable dust and 5 mg/m^3^ for inhalable dust [181]. As an input to limit value discussions in the USA, Wheeler and Bailer [182] applied a model averaging method to evaluate inhalation rat studies with TiO_2_ exposures. The model average estimate of the working lifetime mean respirable dust concentration of TiO_2_ associated with a 1/1000 excess risk of lung cancer was estimated as 9.0 mg/m^3^ [183]. In contrast, CB exposures are measured as inhalable fractions at the work places in US and Canadian production plants according to the interpretations of epidemiological studies and decisions of the US TLV committee. We note that procedures are available to estimate threshold values from epidemiological or toxicological data [184,185] and these methods should be applied more often to derive limit value proposals and to discuss which metric is appropriate.

## Conclusions

We conclude that the problems noted in estimating a HEC by extrapolating overload results of rats to humans need to be addressed to ensure that OELs are based on appropriate scientific assumptions and metrics. Furthermore, any method proposed should be reproducible by other scientists to ensure the accuracy and reliability of the results, especially when used for public policy such as setting OELs.

## Endnotes

^a^Prof. Hartwig is chair of the MAK Commission and Editor of the MAK documents.

^b^The main arguments were made available to the MAK Commission in written form in 2011 and all arguments were publicly presented and discussed with representatives of the MAK Commission at the symposium on the new general dust limit value proposal of the MAK Commission organized by the Committee on Hazardous Substances (Ausschuss für Gefahrstoffe, AGS) of the Federal Ministry of Labour and Social Affairs, held in Dortmund, Germany on April 8, 2013 (http://www.baua.de/de/Themen-von-A-Z/Gefahrstoffe/AGS/AGS-publik-2013.html).

^c^The PF&T Editors recommended, after a pre-submission review of this manuscript, that we contact Prof. Pauluhn to seek clarification from him on a number of technical issues. To this end, we requested from Prof. Pauluhn a copy of the Fortran Code and also provided Prof. Pauluhn with a number of technical questions regarding input data and other details of modelling that were not fully explained in his publication [14]. These details were important as the model was used in the MAK Commission’s GBS Document. Prof. Pauluhn responded to our query, but he did not provide the requested information, partly because of contractual reasons with his previous employer.

## Abbreviations

ACGIH®: American Conference of Governmental Industrial Hygienists
AF: Adaptation factor
ALOOH: Aluminum oxyhydroxides = boehmite
AM: Alveolar macrophage
AOP: Adverse outcome pathway
BAL: Bronchoalveolar lavage
BALF: Bronchoalveolar lavage fluid
BALF-PMNs: PMNs in BALF
BaSO_4_: Barium sulfate
BET: Brunauer-Emmett-Teller method
CB: Carbon black
CS: Crystalline silica
COPD: Chronic obstructive pulmonary disease
DNA: Desoxyribonucleic acid
ECETOC: European Center for Toxicology and Chemicals
ECHA: European Chemicals Agency
Fe_3_O_4_: Magnetite
FRC: Functional residual capacity
GBS: Granular biopersistent dusts (granuläre biobeständige Stäube)
GM: Geometric mean
GSD: Geometric standard deviation
GSH: Glutathione
H: Hamster
HEC: Human equivalent concentration
IA: Inhalability adjustment
ICBA: International Carbon Black Association
ICRP: International Commission on Radiation Protection
ILD: Interstitial lung diseases
IOM: Institute of Occupational Medicine, Edinburgh, UK
LSLTP: Low-solubility low-toxicity particles
M: Mouse
MAK: MAK limit value (Maximale Arbeitsplatz-Konzentration)
MIP 2: Macrophage inflammatory protein 2
MLE: Maximum likelihood estimates
MMAD: Mass median aerodynamic diameter
MoA: Mode of action
MPPD: Multiple-path particle dosimetry model
Nano-BaSO_4_: Nanostructured BaSO_4_
NOAEC: No-observed adverse-effect concentration
NOAEL: No observed adverse-effect level
OEL: Occupational exposure limit
OMB: Oronasal-mouth breather
ONA: Oronasal-normal augmenter
PAH: Polycyclic aromatic hydrocarbon
PG: Phosphatidylglycerol
PI: Phosphatidylinitisol
PM_resp_: Deposition fraction in %
PMF: Progressive massive fibrosis
PMN: Polymorphonuclear neutrophil
PNOC: Particles not otherwise classified
R: Rat
RNS: Reactive nitrogen species
ROS: Reactive oxygen species
SCOEL: Scientific Committee on Occupational Exposure Limits
SOD: Superoxide dismutase
TiO_2_: Titanium dioxide
TLC: Total lung capacity
TLV®: Threshold limit value
TNF alpha: Tumour necrosis factor alpha
TWA: Time-weighted average
U.S. EPA: US Environmental Protection Agency
US TLV: US threshold limit value
8-oxoGua: 8-oxo-7,8-dihydro-2′-deoxiguanosine
